# Supplementary figures and images for: Assessing landscape aesthetic values: Do clouds in photographs influence people’s preferences?
Source: PLoS One. 2023 Jul 28;18(7):e0288424. doi: 10.1371/journal.pone.0288424 (PMC10381034; doi:10.1371/journal.pone.0288424)

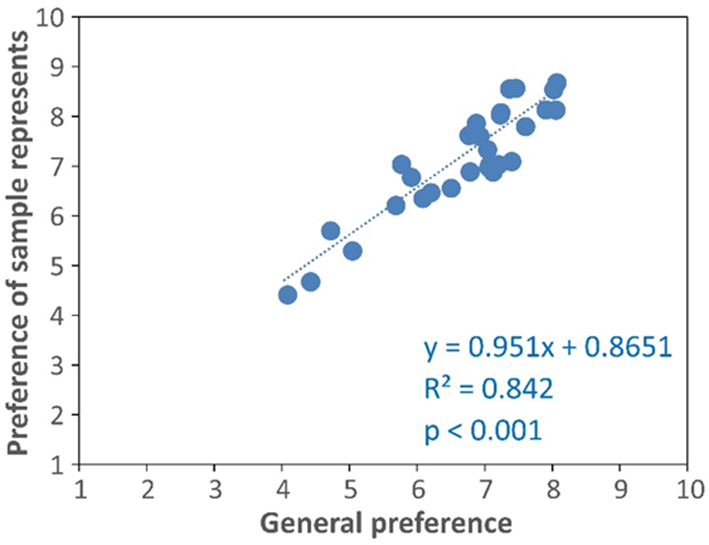

Supplement: S2 Fig — (TIF) [file pone.0288424.s002.tif]

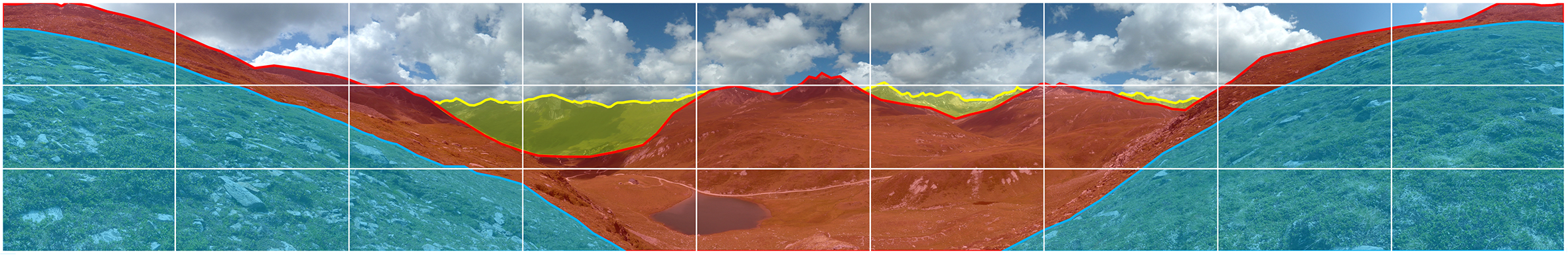

Supplement: S4 Fig — Blue = near zone (< 60 m), red = middle zone (> 60 m– 1.5 km), yellow = far zone (>1.5 km), and auxiliary grids to determine the area fraction of distance zones and landscape features. Own photograph. (TIF) [file pone.0288424.s004.tif]

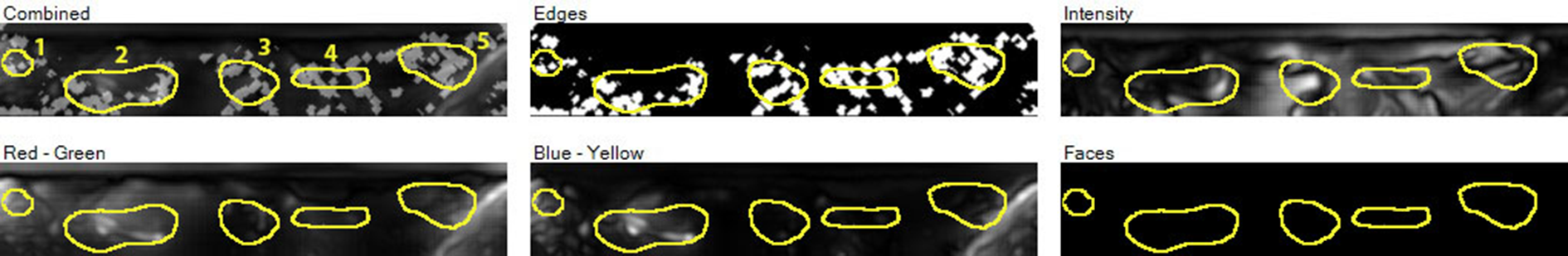

Supplement: S5 Fig — The yellow circles indicate the hotspots identified in 3M-VAS. Within each hotspot, the importance of each visual element was estimated on a scale of from 0 to 100%. For example, for hotspot no. 3, edges take up 50% of the area, intensity has mostly medium to high values (grey to light grey areas), red-green contrast values are low (dark grey patterns) and blue-yellow contrasts as well as no values for faces (black) are missing. Estimated contributions are 50% for edges, 40% for intensity, 5% for red-green color contrast and 0% for blue-yellow color contrast and faces. Own photograph. (TIF) [file pone.0288424.s005.tif]

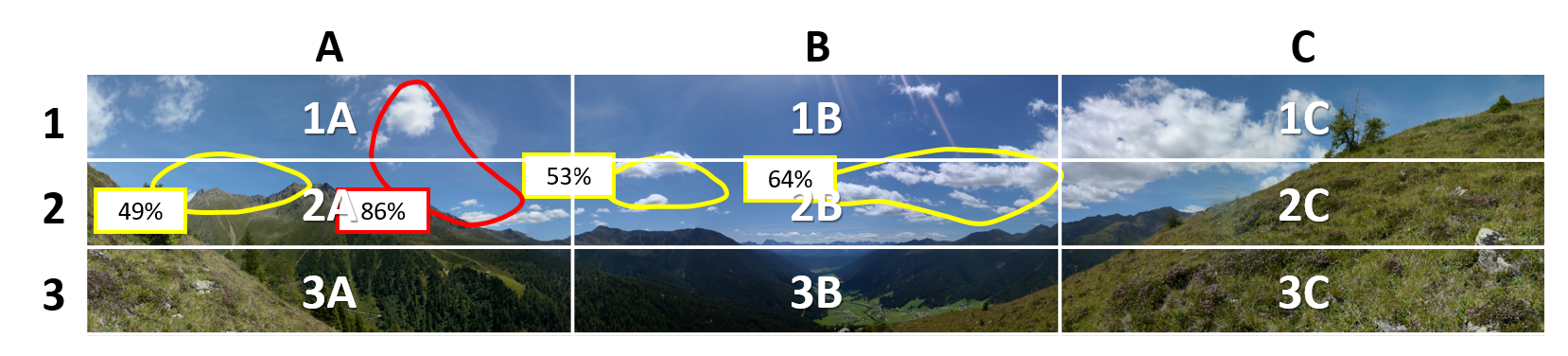

Supplement: S6 Fig — This grid was used to determine a spatial shift between pictures, for which the preference scores significantly differed between the original picture with clouds and the manipulated picture without clouds. Own photograph. (TIF) [file pone.0288424.s006.tif]
