# Supplementary material for: Assessing landscape aesthetic values: Do clouds in photographs influence people’s preferences?
Source: PLoS One. 2023 Jul 28;18(7):e0288424. doi: 10.1371/journal.pone.0288424 (PMC10381034; doi:10.1371/journal.pone.0288424)

Picture 1

A

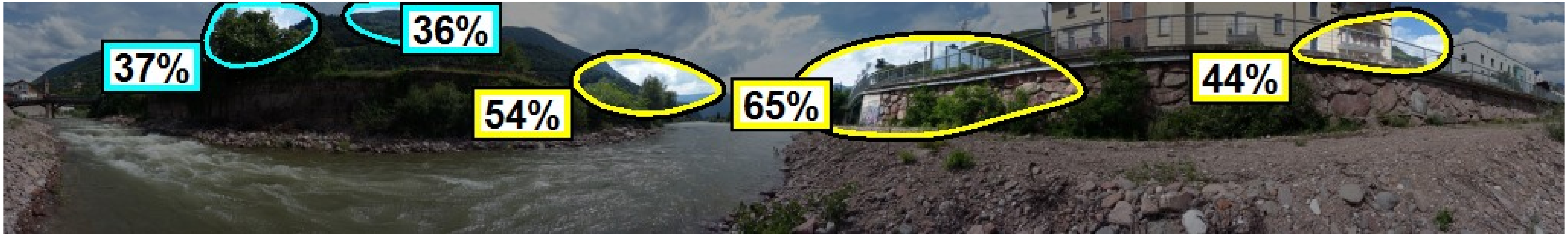

B

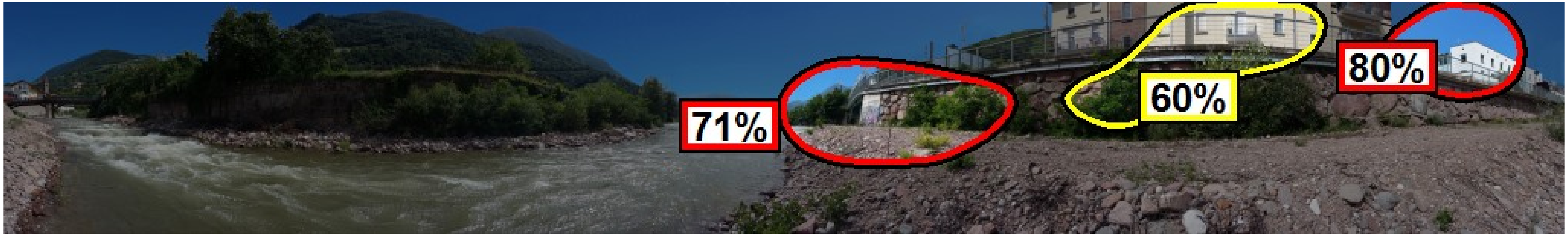

Picture 2

A

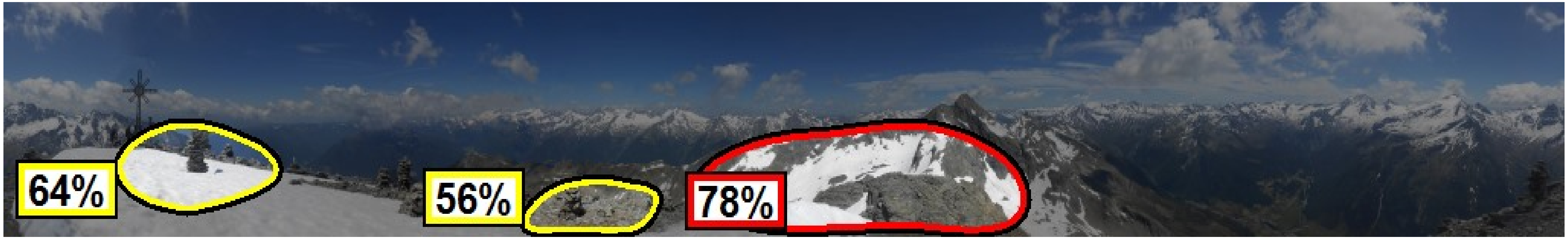

B

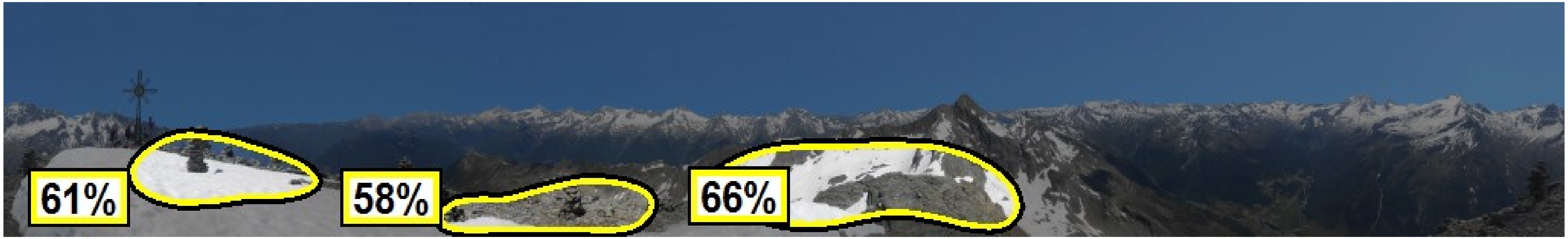

Picture 3

A

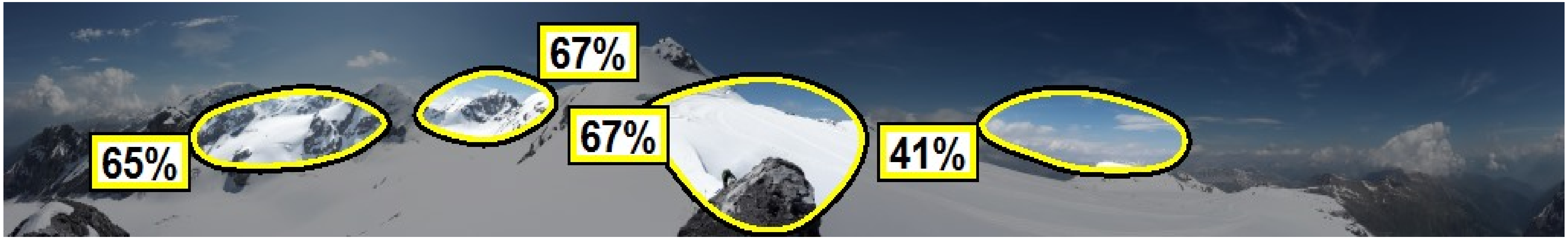

B

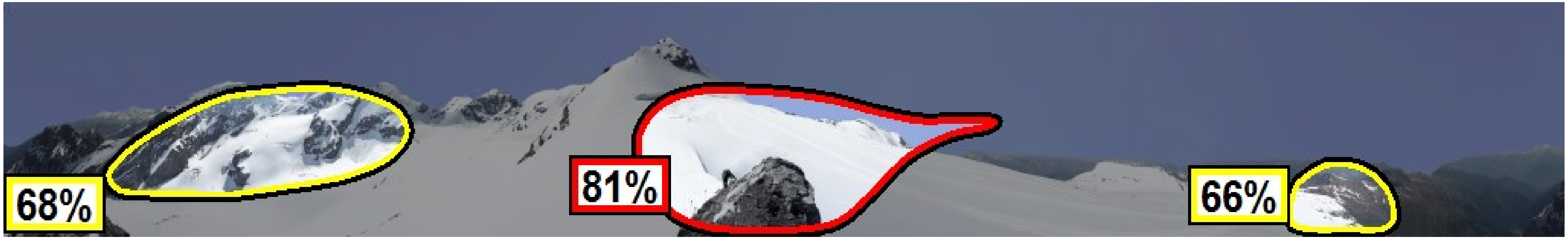

Picture 4

A

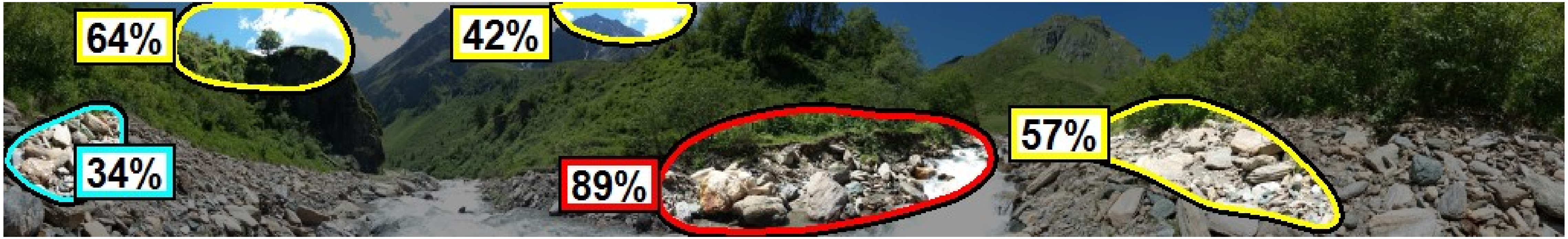

B

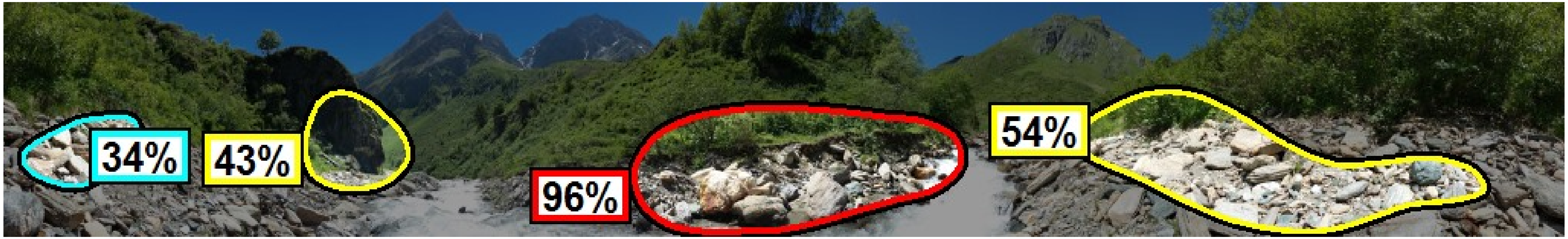

Picture 5

A

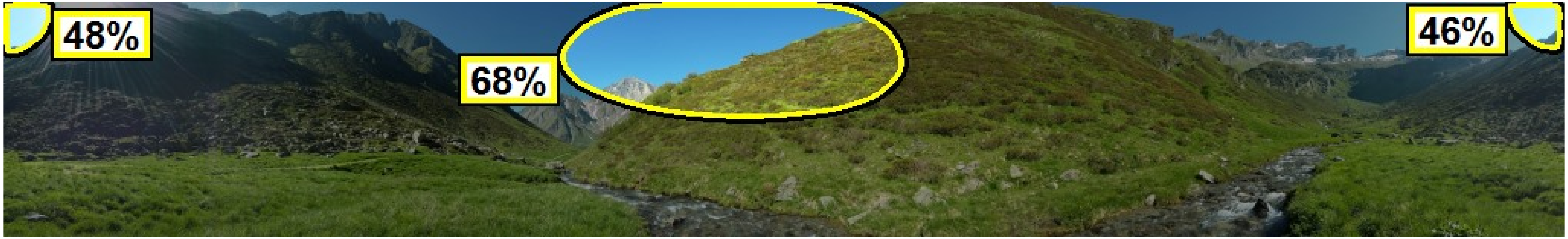

B

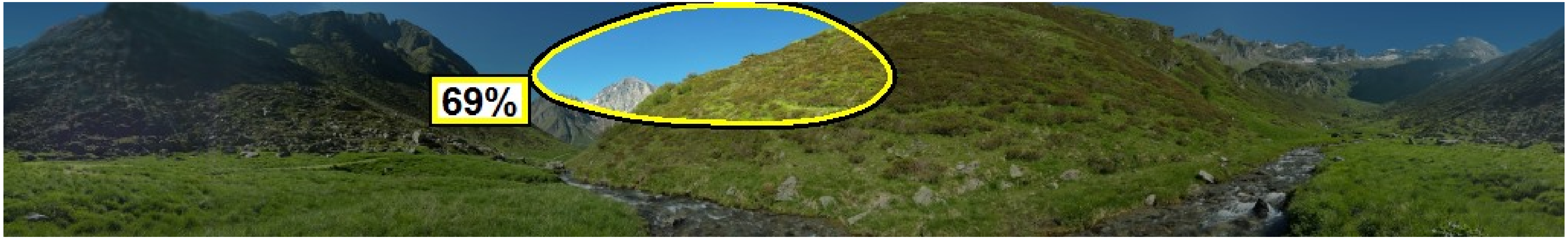

Picture 6

A

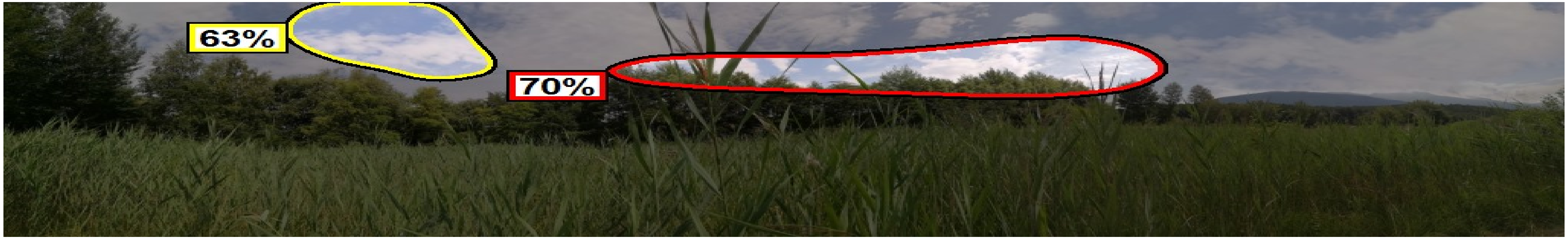

B

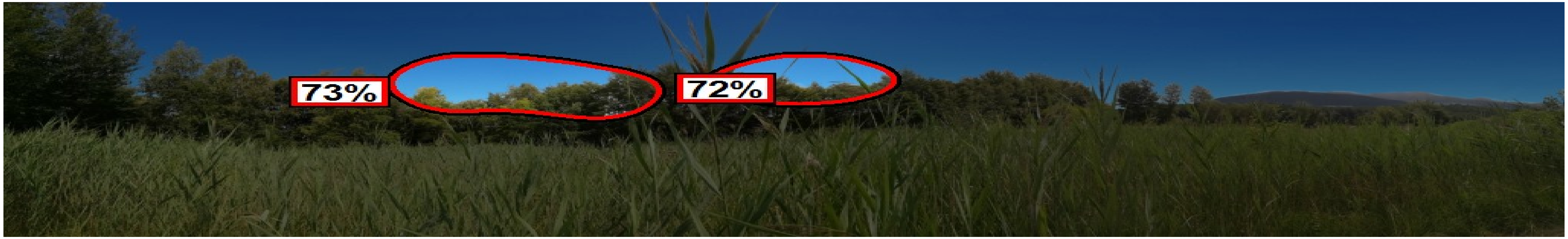

Picture 7

A

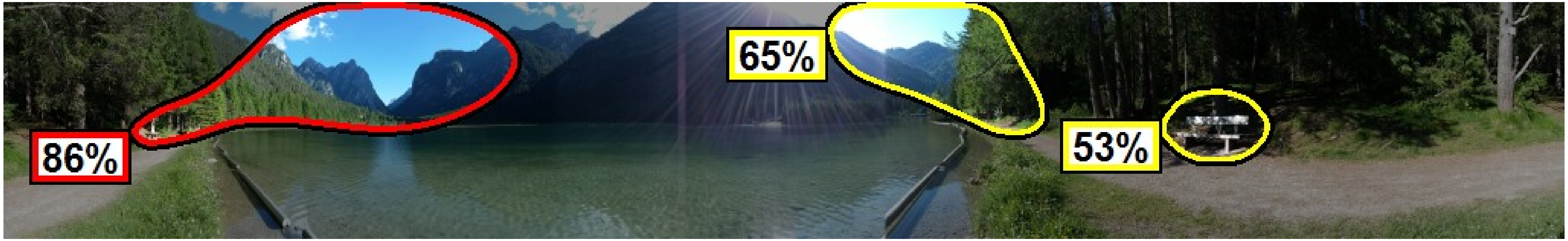

B

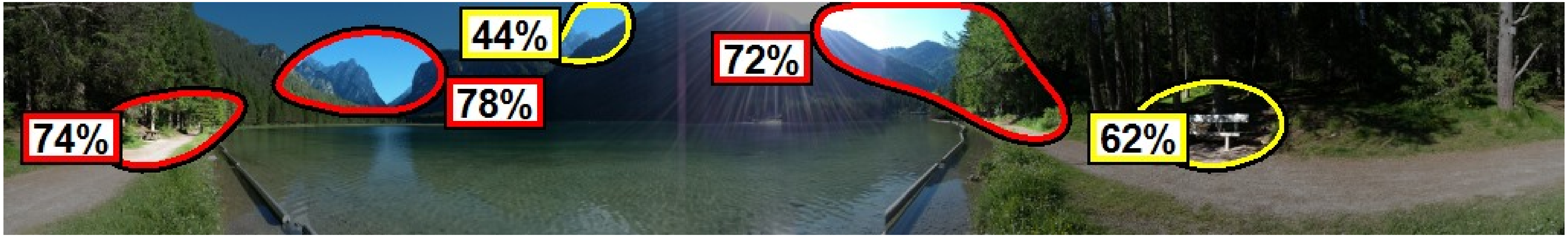

Picture 8

A

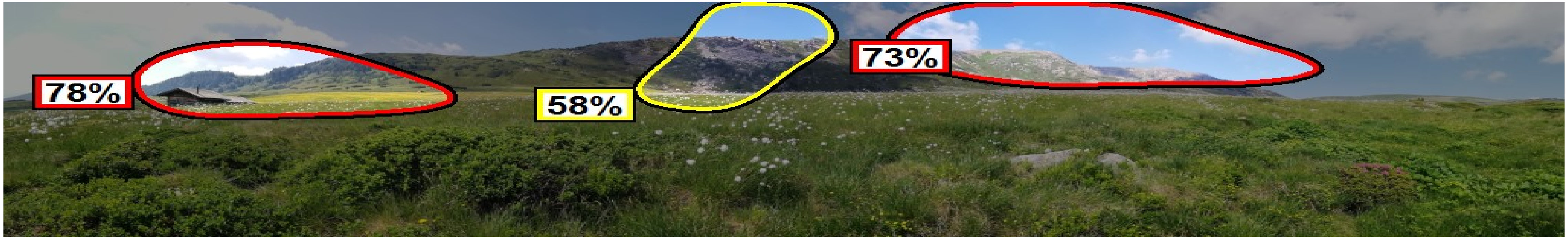

B

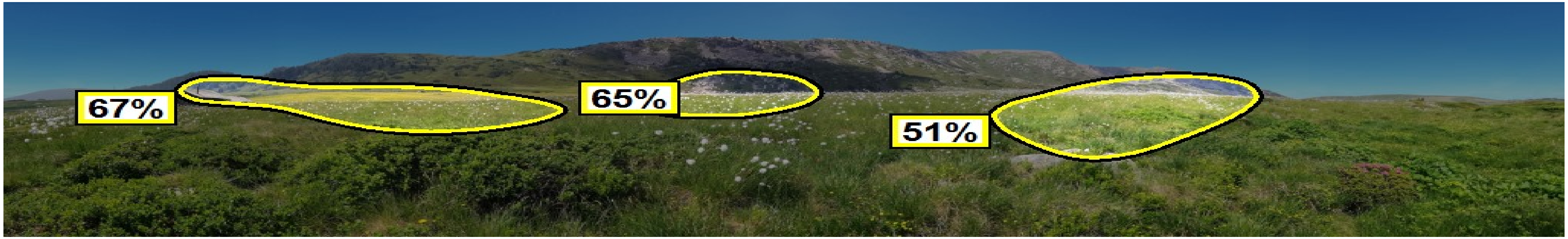

Picture 9

A

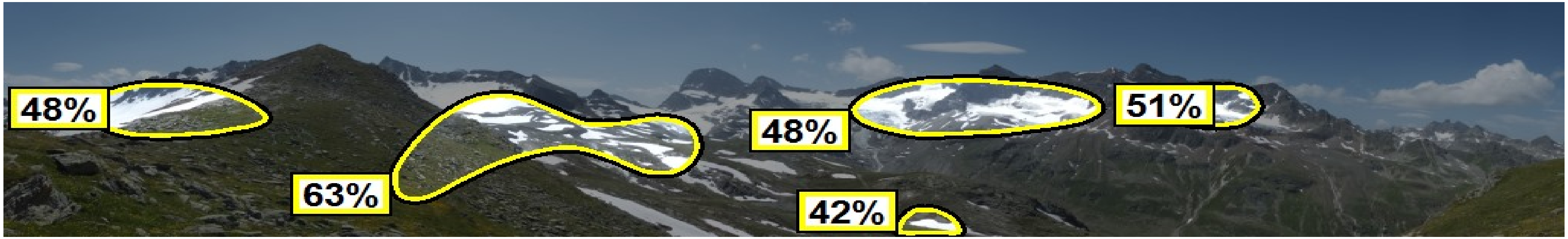

B

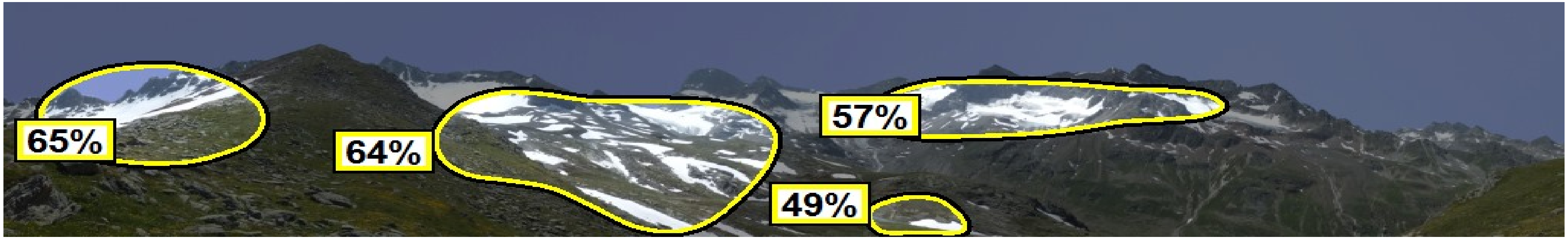

Picture 10

A

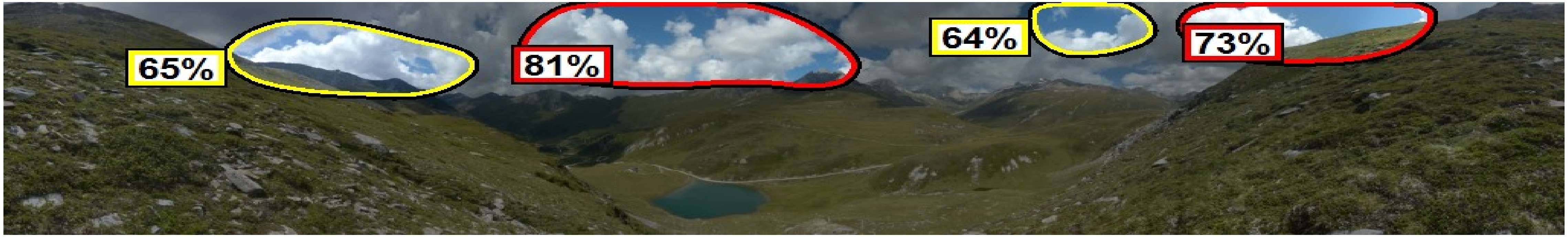

B

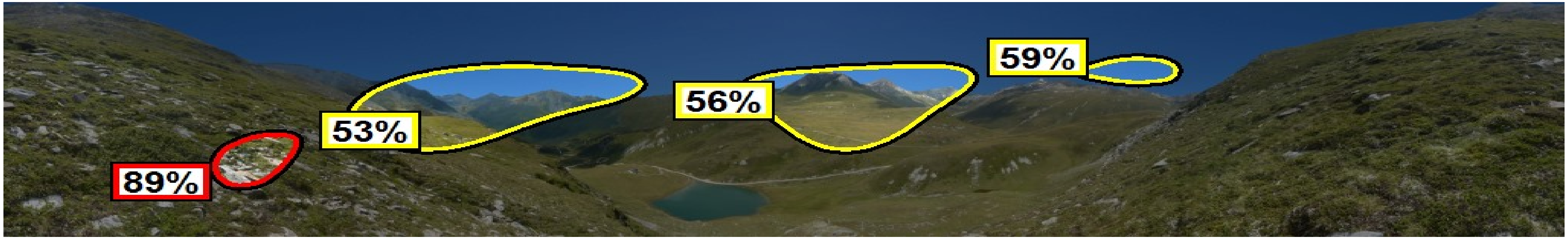

Picture 11

A

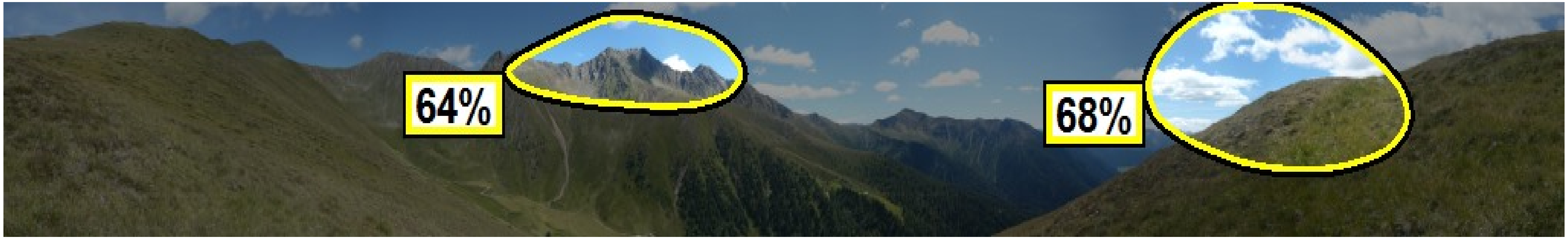

B

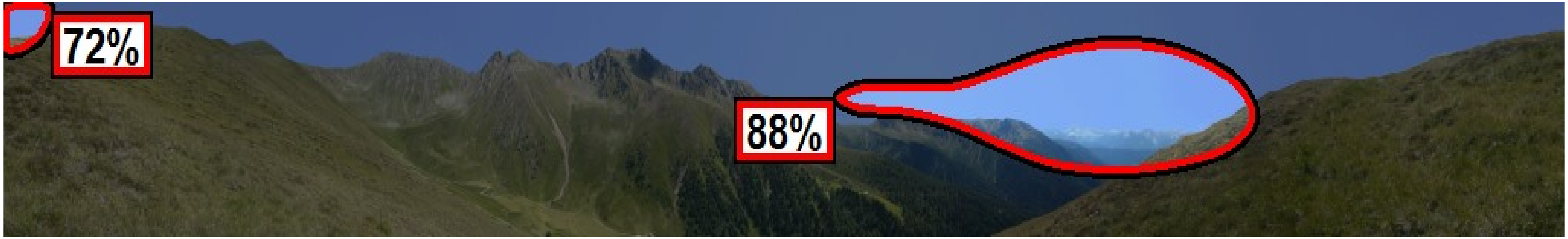

Picture 12

A

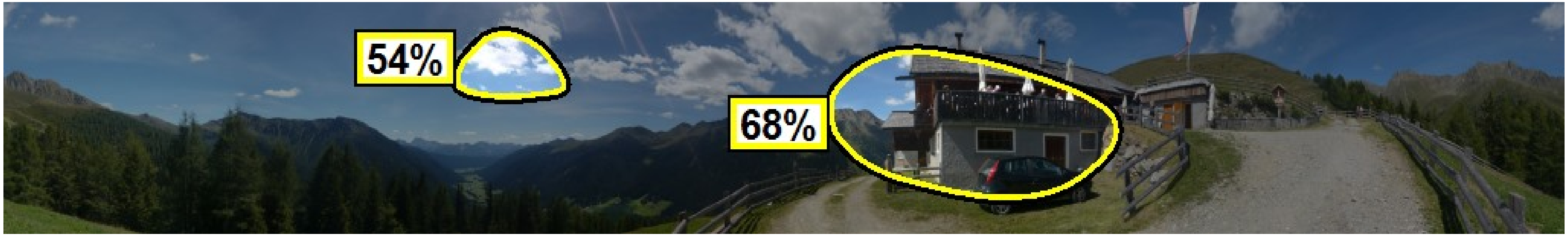

B

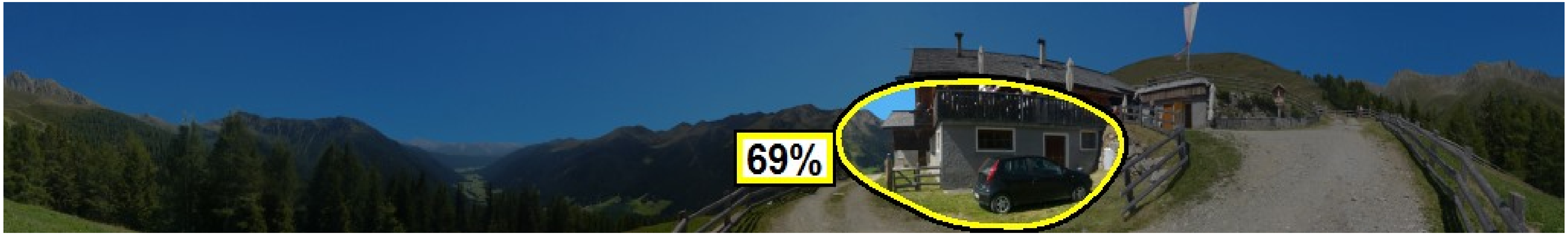

Picture 13

A

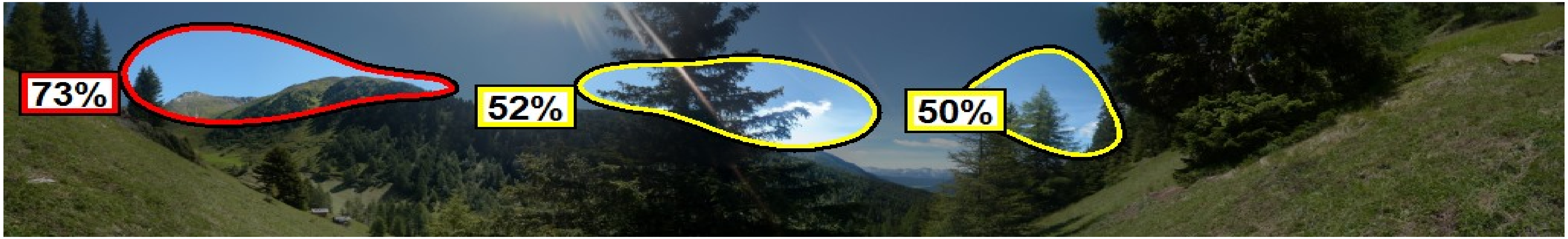

B

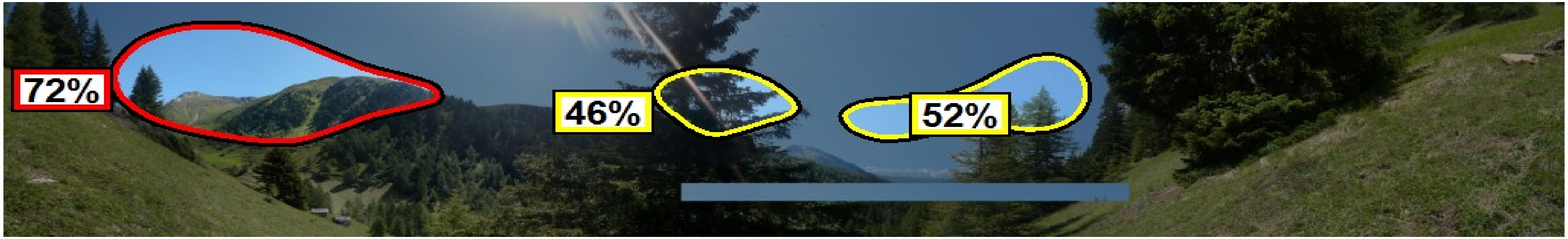

Picture 14

A

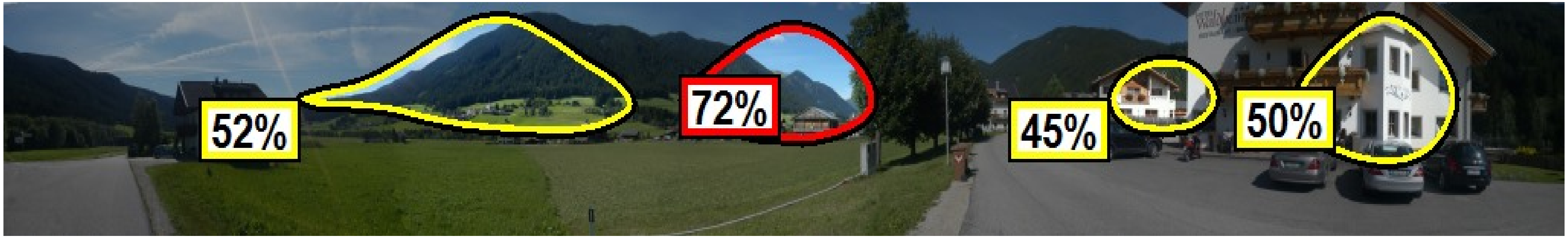

B

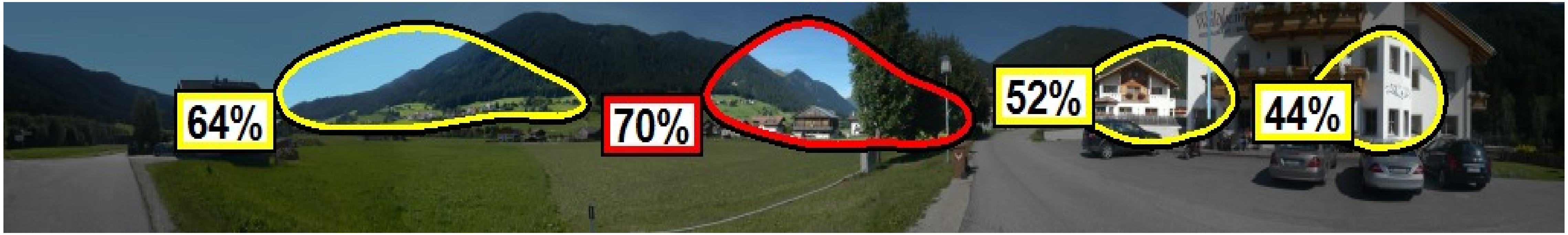

Picture 15

A

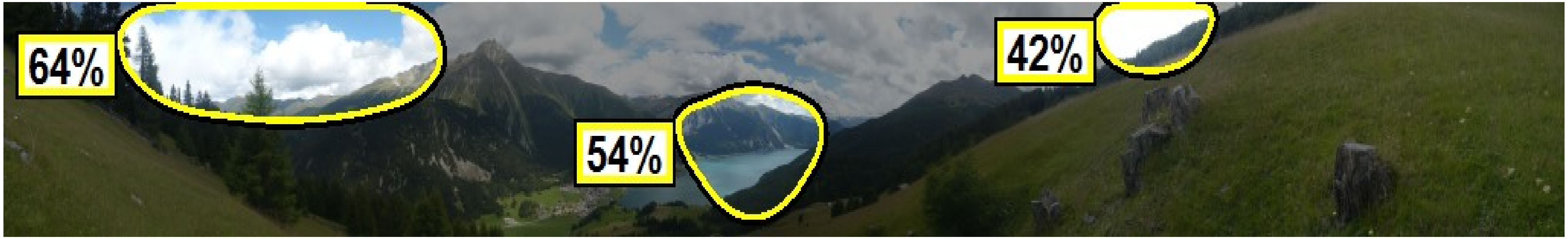

B

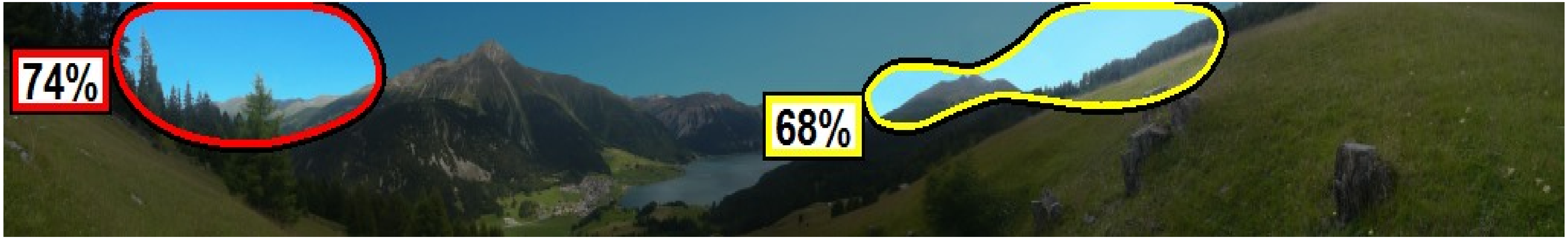

Picture 16

A

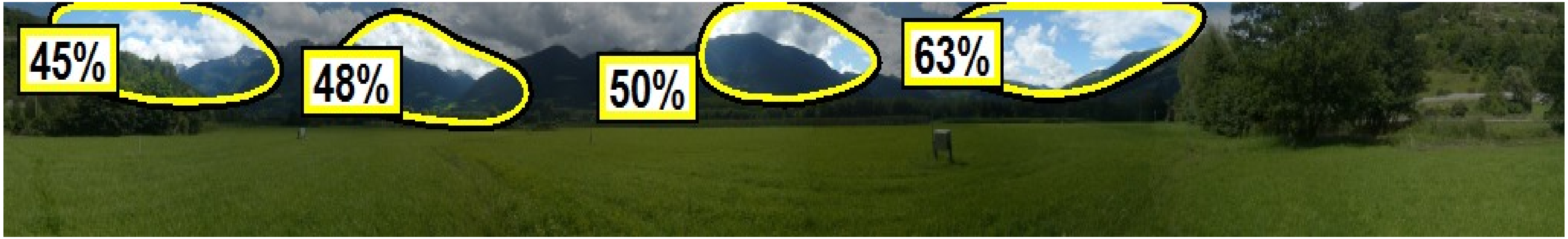

B

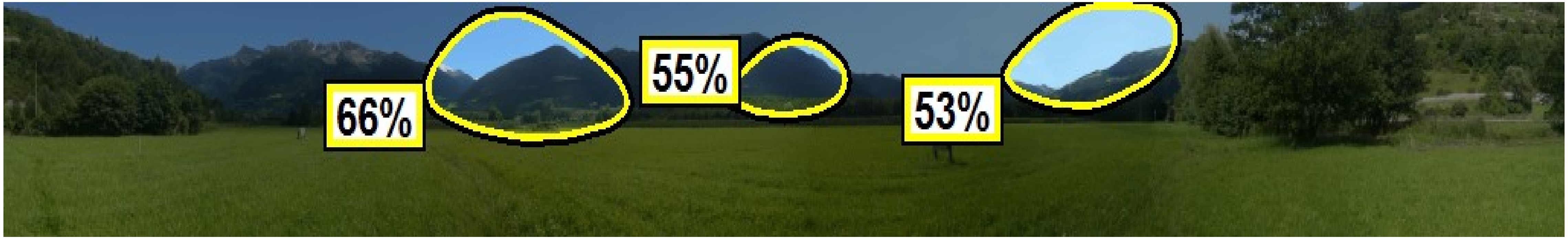

Picture 17

A

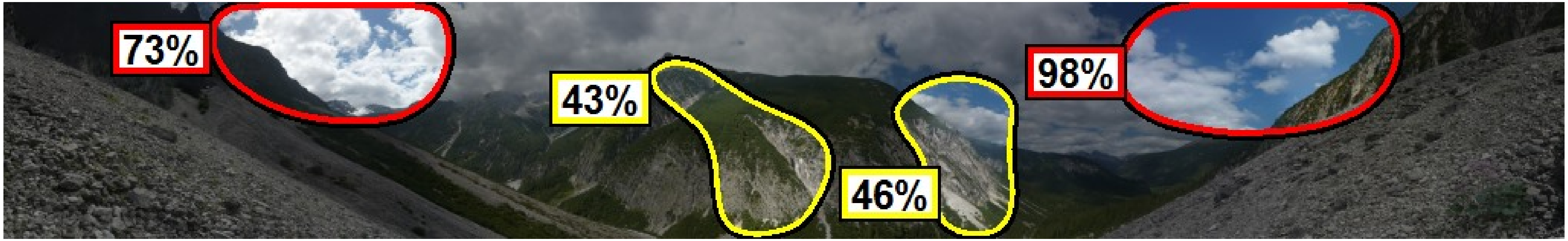

B

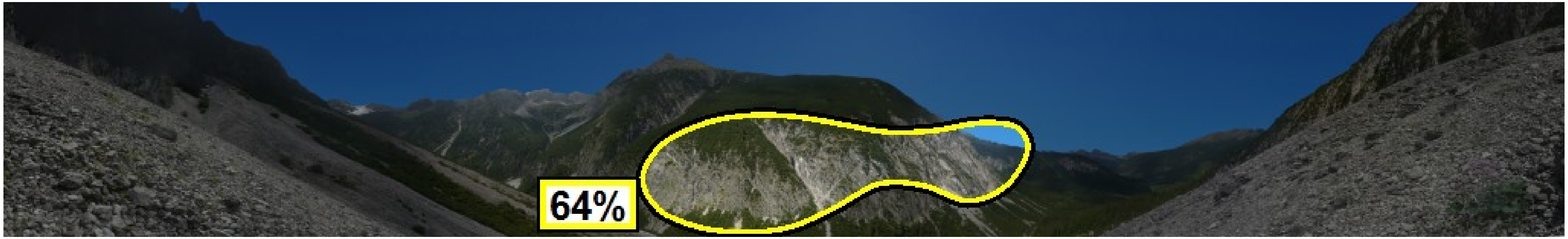

Picture 18

A

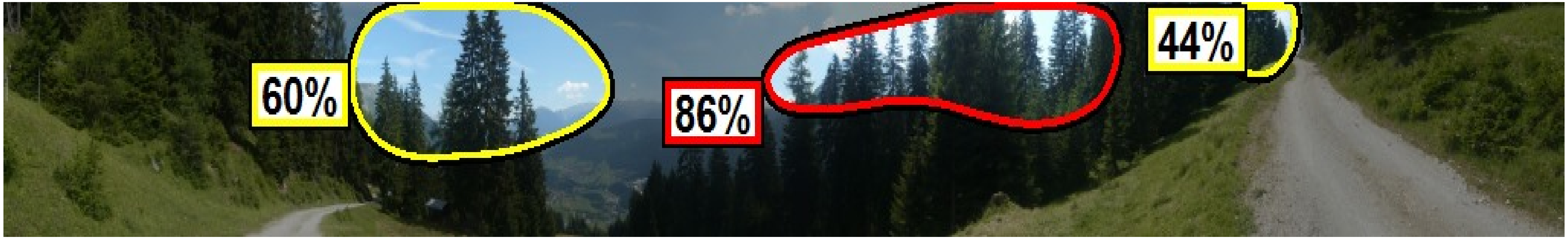

B

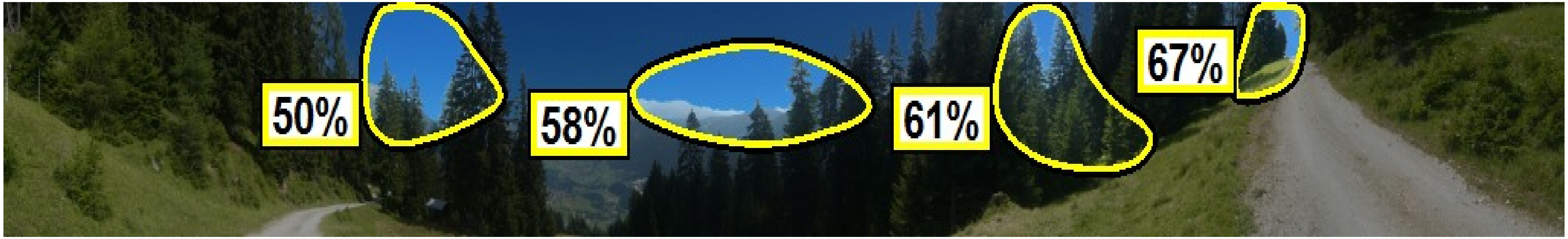

Picture 19

A

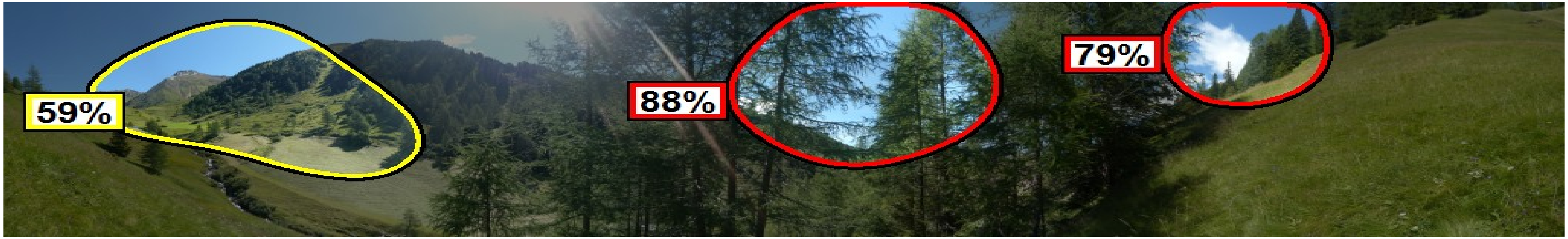

B

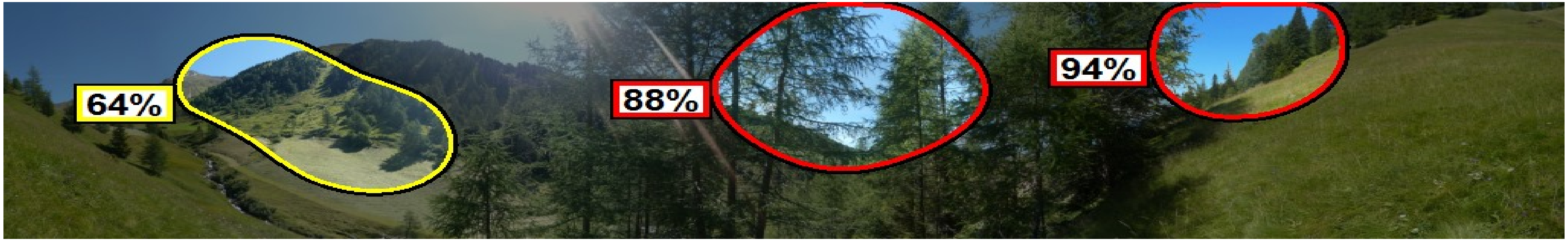

Picture 20

A

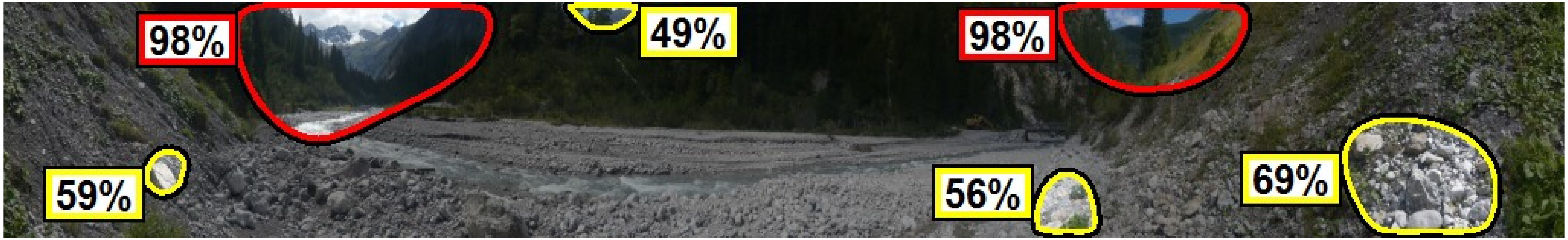

B

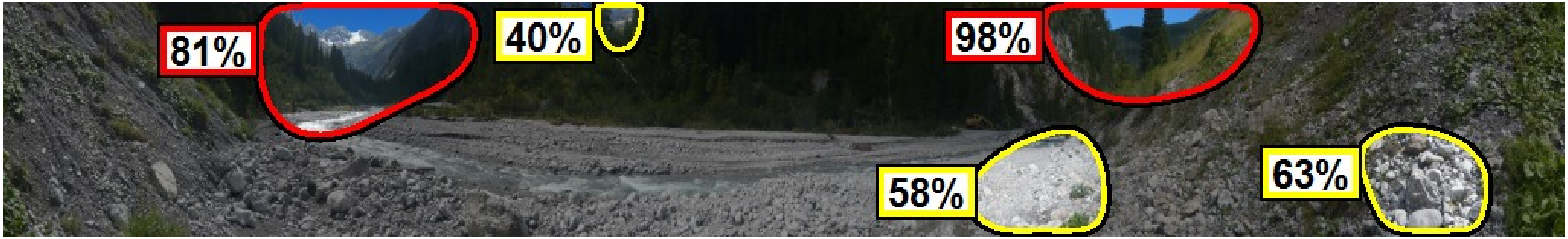

Picture 21

A

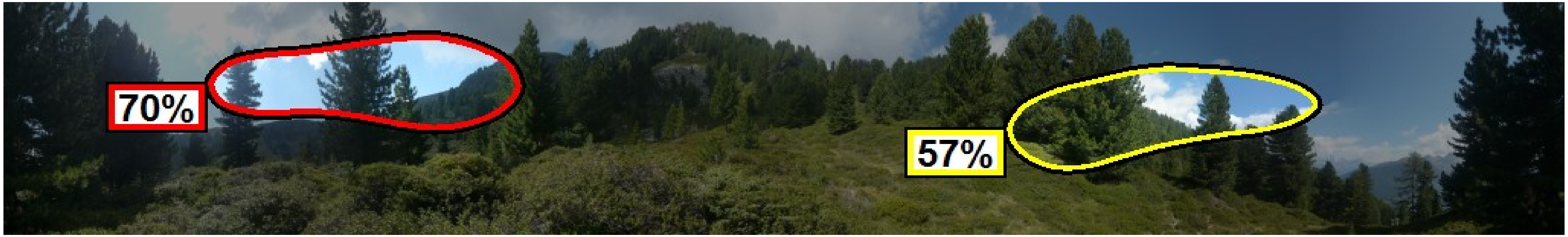

B

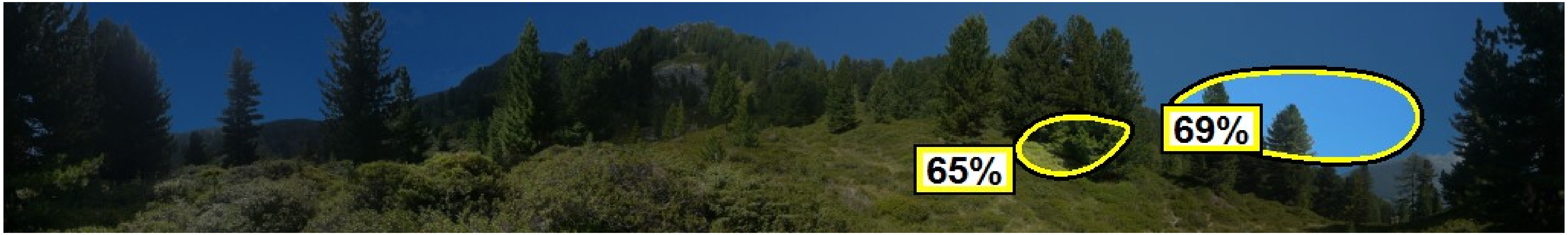

Picture 22

A

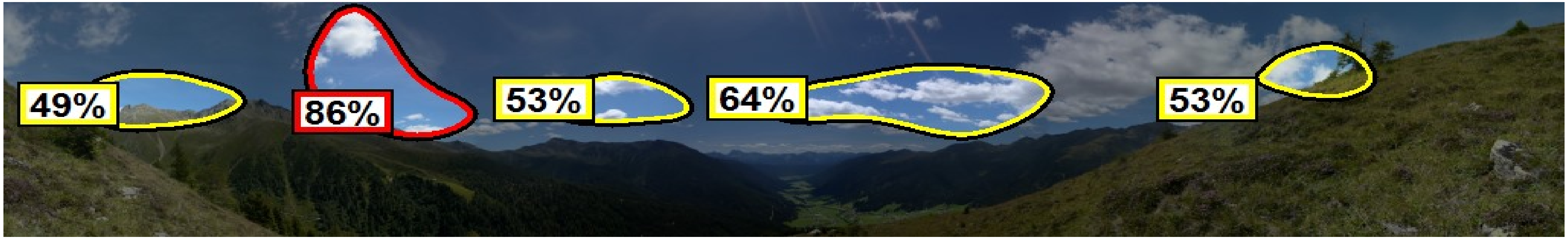

B

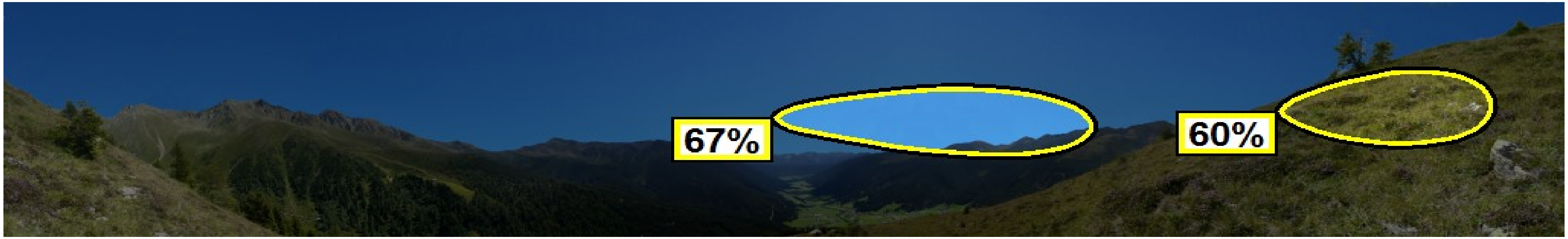

Picture 23

A

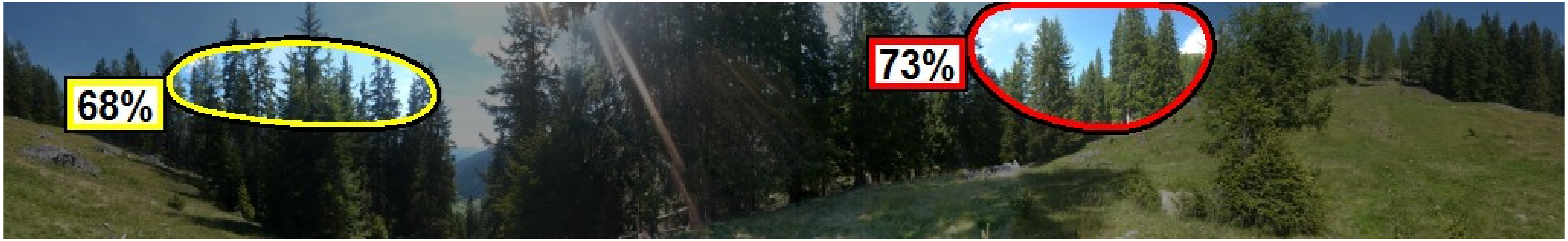

B

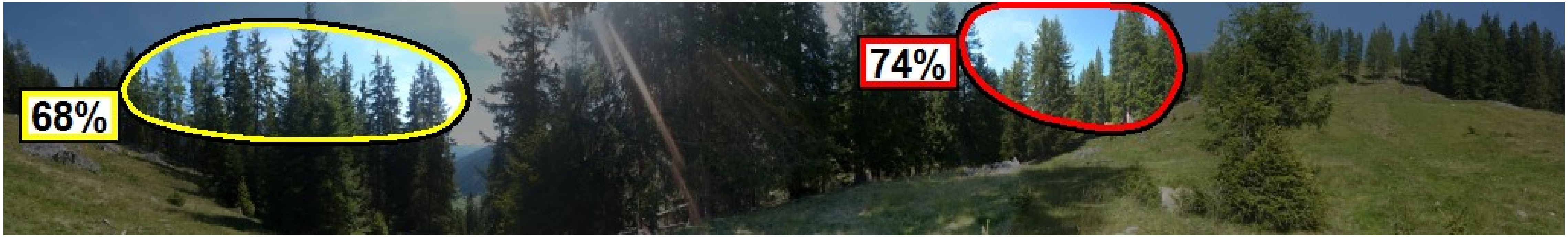

Picture 24

A

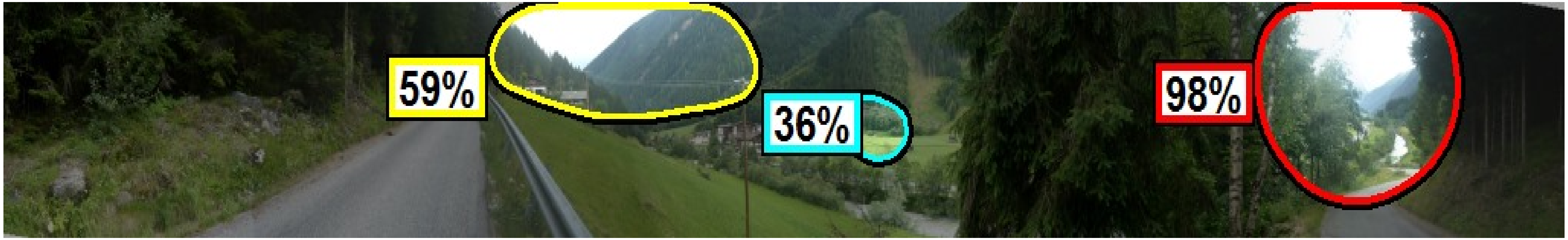

B

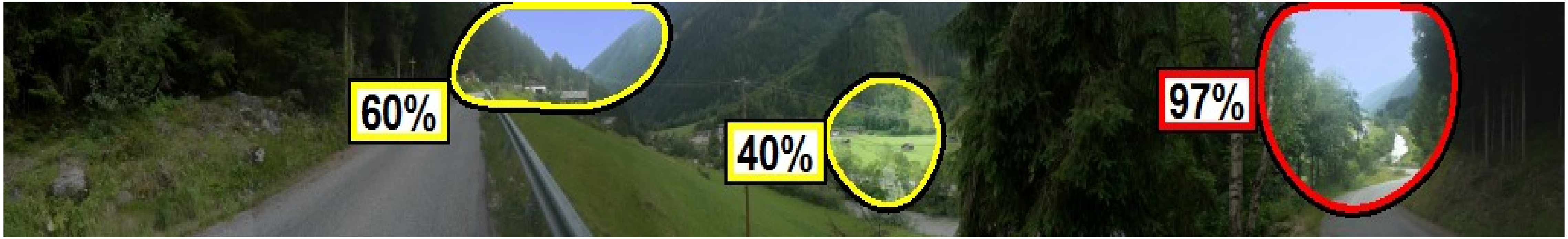

Picture 25

A

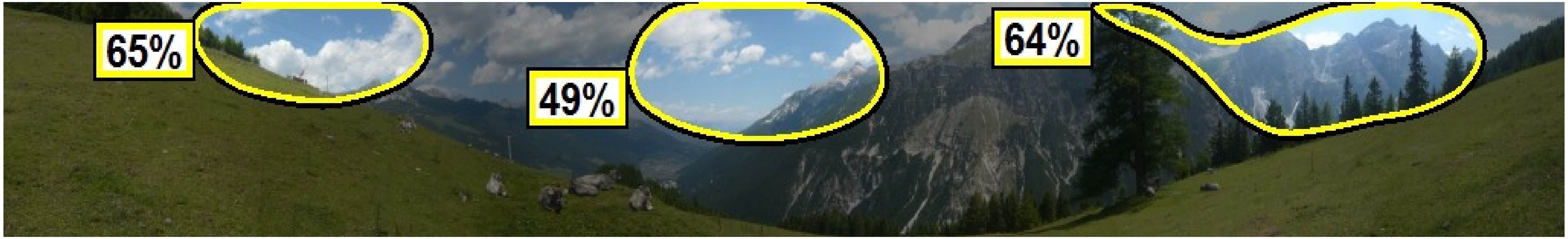

B

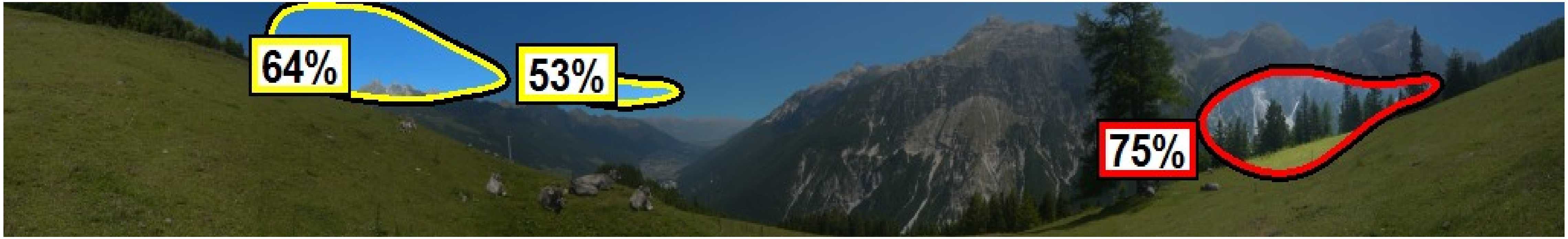

Picture 26

A

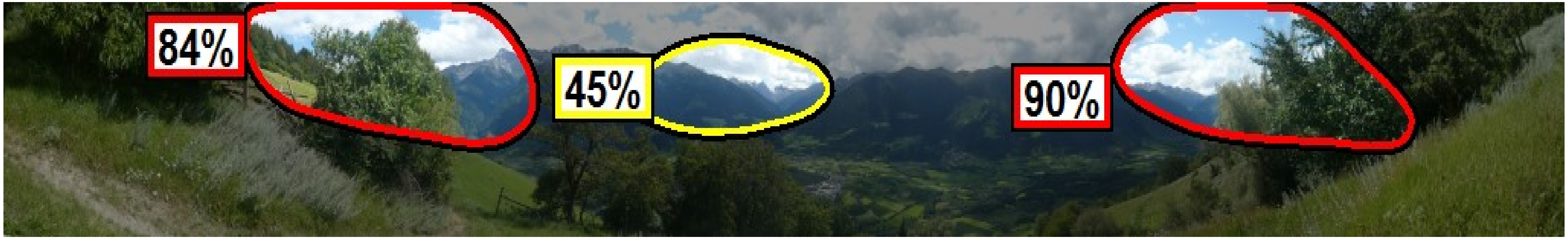

B

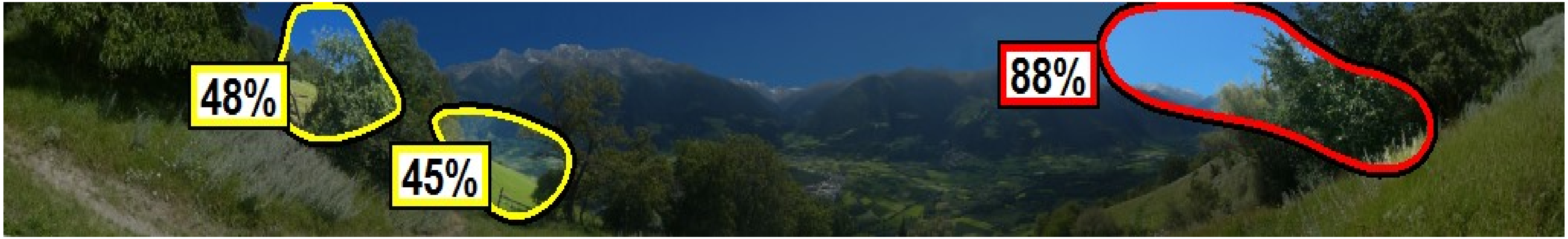

Picture 27

A

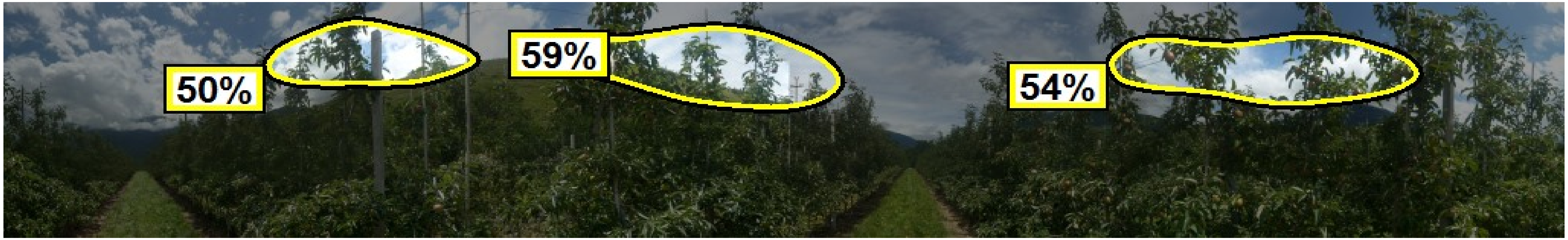

B

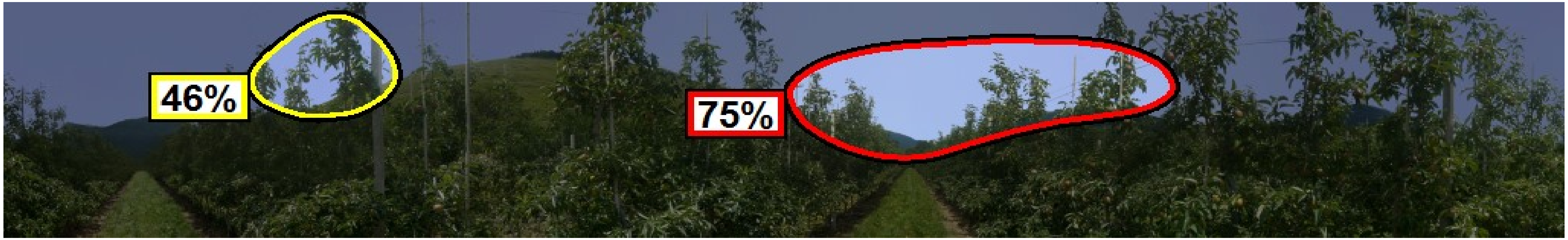

Picture 28

A

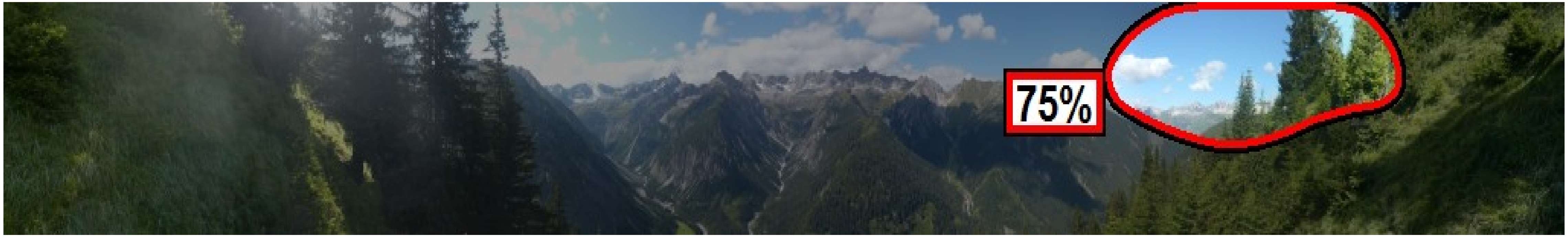

B

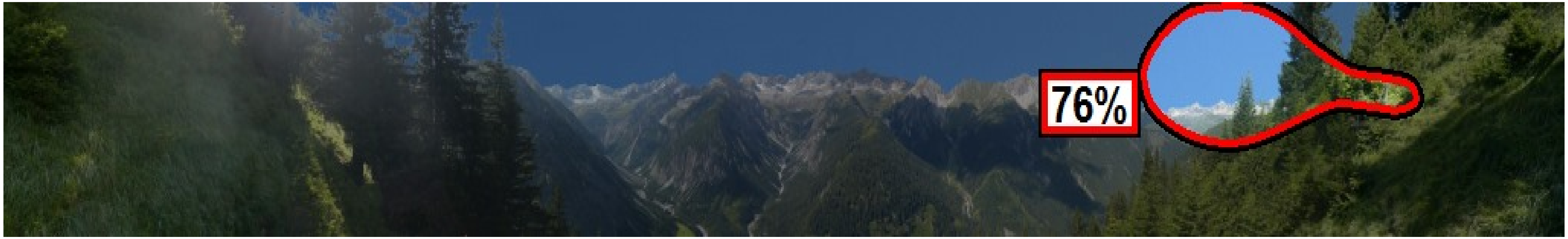

Picture 29

A

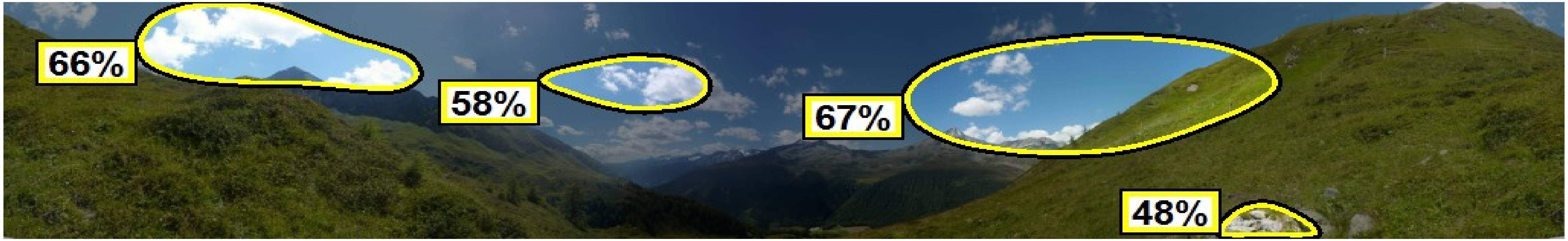

B

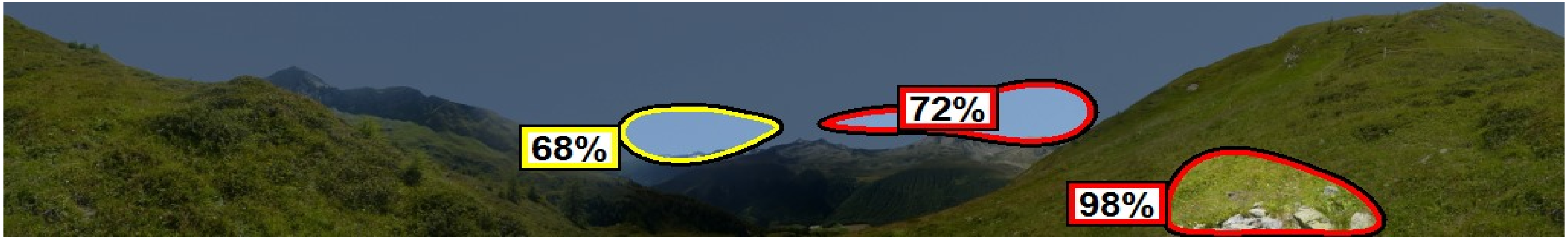

Supplement: S3 Fig — Hotspots in original photographs (A) and manipulated pictures (B) derived from eye-tracking simulation using 3M-VAS. Own photographs. (PDF) [file pone.0288424.s003.pdf]
