# Supplementary material for: Assessing landscape aesthetic values: Do clouds in photographs influence people’s preferences?
Source: PLoS One. 2023 Jul 28;18(7):e0288424. doi: 10.1371/journal.pone.0288424 (PMC10381034; doi:10.1371/journal.pone.0288424)
Supplement: S1 Table — This information was not required to complete the questionnaire, and some participants did not at all or only partly indicate demographic details. (DOCX) [file pone.0288424.s007.docx]

Table S1: Demographic information of the survey participants (n=124). This information was not required to complete the questionnaire, and some participants did not at all or only partly indicate demographic details.

| Category | Demographic details | Number of respondents (n) | Percentage (%) |
| --- | --- | --- | --- |
| Gender | Female | 83 | 70,3% |
|  | Male | 34 | 28,8% |
|  | Other | 1 | 0,8% |
| Age | < 25 years | 81 | 66,4% |
|  | 25 - 60 years | 37 | 30,3% |
|  | > 60 years | 4 | 3,3% |
| Language | German | 113 | 92,6% |
|  | Other | 9 | 7,4% |
| Origin | Resident in North Tyrol | 72 | 69,2% |
|  | Resident in South Tyrol | 20 | 19,2% |
|  | Tourist | 12 | 11,5% |
| Living place | Village | 53 | 43,8% |
|  | Town (<= 50,000 inhabitants) | 21 | 17,4% |
|  | City (> 50,000 inhabitants) | 47 | 38,8% |
| Education | Secondary school | 2 | 1,6% |
|  | High school | 85 | 69,7% |
|  | University | 31 | 25,4% |
|  | Other | 4 | 3,3% |
| Working sector | Agriculture/forestry | 2 | 1,7% |
|  | Industry/production | 7 | 5,8% |
|  | Service sector | 35 | 29,2% |
|  | I do not work (yet)/in education | 76 | 63,3% |
| Knowledge on the environment and related issues | Very good | 23 | 19,0% |
|  | Good | 65 | 53,7% |
|  | Sufficient | 25 | 20,7% |
|  | Little | 7 | 5,8% |
|  | No knowledge at all | 1 | 0,8% |
