# Supplementary material for: Assessing landscape aesthetic values: Do clouds in photographs influence people’s preferences?
Source: PLoS One. 2023 Jul 28;18(7):e0288424. doi: 10.1371/journal.pone.0288424 (PMC10381034; doi:10.1371/journal.pone.0288424)
Supplement: S2 Table — (1) Primary hotspot characteristics (i.e., quantitative and spatial information generated by 3M-VAS) and (2) secondary hotspot characteristics (i.e., variables in relation to the different distance zones as well as natural and artificial features within the hotspots). (DOCX) [file pone.0288424.s008.docx]

Table S2: List of all variables derived from eye-tracking simulation as proposed by Schirpke et al. [1]. (1) Primary hotspot characteristics (i.e., quantitative and spatial information generated by 3M-VAS) and (2) secondary hotspot characteristics (i.e., variables in relation to the different distance zones as well as natural and artificial features within the hotspots).

| **Group** | **Variable** | **Unit** | **Description** |
| --- | --- | --- | --- |
| (1) | Number of hotspots | n | Number of hotspots within the photo |
|  | Hotspot area | % | Total estimated area of the hotspots in the photo |
|  | Mean hotspot probability | % | Mean probability of initially looking at the hotspots, mean across all hotspots |
|  | Area-weighted mean hotspot probability | % | Area-weighted mean probability of initial eye-tracking movement, mean of all hotspots |
|  | Highest hotspot probability | % | Probability of initial eye-tracking movement of the hotspot with the highest probability (top-hotspot) |
|  | Lowest hotspot probability | % | Probability of initial eye-tracking movement of the hotspot with the lowest probability |
|  | Top-hotspot area | % | Estimated area of the top-hotspot |
| (2) | Hotspots near | n | Number of hotspots within the near zone (<60 m) |
|  | Hotspots middle | n | Number of hotspots within the middle zone (0.06-1.5 km) |
|  | Hotspots far | n | Number of hotspots within the far zone (>1.5 km) |
|  | Sky hotspots | % | Estimated area of sky within the hotspots |
|  | Clouds hotspots | % | Estimated area of clouds within the hotspots |
|  | Light hotspots | % | Estimated area of special lighting phenomena within the hotspots |
|  | Snow hotspots | % | Estimated area of snow/glacier within the hotspots |
|  | Natural elements hotspots | % | Estimated area of individual natural elements in the foreground within the hotspots (e.g., stones, flowers, plants) |
|  | Artificial elements hotspots | % | Estimated area of individual artificial elements in the foreground within the hotspots (e.g., streets, street signs, cars, fences) |
|  | Edges of visual elements | % | Estimated mean contribute of edges of visual elements within the hotspots to the overall probability of the hotspot (Edges_3MVAS) |
|  | Intensity of visual elements | % | Estimated mean contribute of intensity of visual elements (i.e., luminance contrast, brightness, black/white contrast) within the hotspots to the overall probability of the hotspot (Intensity_3MVAS) |
|  | Red-green color contrast | % | Estimated mean contribute of red-green color contrast of visual elements within the hotspots to the overall probability of the hotspot (Redgreen_3MVAS) |
|  | Blue-yellow color contrast | % | Estimated mean contribute of blue-yellow color contrast of visual elements within the hotspots to the overall probability of the hotspot (Blueyellow_3MVAS) |

1. Schirpke U, Tasser E, Lavdas AA. Potential of eye-tracking simulation software for analyzing landscape preferences. PLOS ONE. 2022;17: e0273519. Available: https://doi.org/10.1371/journal.pone.0273519
