# Supplementary material for: Assessing landscape aesthetic values: Do clouds in photographs influence people’s preferences?
Source: PLoS One. 2023 Jul 28;18(7):e0288424. doi: 10.1371/journal.pone.0288424 (PMC10381034; doi:10.1371/journal.pone.0288424)
Supplement: S3 Table — (DOCX) [file pone.0288424.s009.docx]

Table S3: List of all variables derived from photo content analysis as proposed by Schirpke et al. [1].

| **Variable** | **Unit** | | **Description** |  |
| --- | --- | --- | --- | --- |
| Distance zones | | n | Number of distance zones in the photo (near zone (0-60 m), middle zone (0.06-1.5 km), far zone (>1.5 km) | |
| Percentage near zone | | % | Estimated near zone (0-60 m) within the photo | |
| Percentage middle zone | | % | Estimated middle zone (0.06–1.5 km) within the photo | |
| Percentage far zone | | % | Estimated far zone (>1.5 km) within the photo | |
| Natural elements | | % | Estimated area of clearly recognizable natural elements within photo (e.g., stones, flowers, single plants) | |
| Artificial elements | | % | Estimated area of clearly recognizable artificial elements within the photo (e.g. street, street signs, cars, fences) | |
| Sky | | % | Estimated area of sky within the photo | |
| Clouds | | % | Estimated area of clouds within the sky | |
| Light | | % | Estimated area of special lighting conditions within the photo | |
| Open soil | | % | Estimated area of open soil cover | |

1. Schirpke U, Tasser E, Lavdas AA. Potential of eye-tracking simulation software for analyzing landscape preferences. PLOS ONE. 2022;17: e0273519. Available: https://doi.org/10.1371/journal.pone.0273519
