# Supplementary material for: Assessing landscape aesthetic values: Do clouds in photographs influence people’s preferences?
Source: PLoS One. 2023 Jul 28;18(7):e0288424. doi: 10.1371/journal.pone.0288424 (PMC10381034; doi:10.1371/journal.pone.0288424)
Supplement: S4 Table — (DOCX) [file pone.0288424.s010.docx]

Table S4: List of all variables derived from Geographic Information System (GIS)-based analysis as proposed by Schirpke et al. [1].

| **Variable** | **Acronym** | **Unit** |
| --- | --- | --- |
| Area-weighted mean patch area distribution | AREA_AM | ha |
| Standard deviation in related circumscribing circle distribution | CIRCLE_SD | - |
| Median of contiguity index | CONTIG_MD | - |
| Median of gyration radius | GYRATE_MD | m |
| Largest patch index | LPI | - |
| Modified Simpson's diversity index | MSIDI | - |
| Number of patches | NP | n |
| Patch density | PD | n km^-2^ |
| Patch richness | PR | - |
| Standard deviation shape index | SHAPE_CV | - |
| Area near zone (<1.5 km) | TA_1 | ha |
| Area middle zone (1.5-10 km) | TA_2 | ha |

1. Schirpke U, Tasser E, Lavdas AA. Potential of eye-tracking simulation software for analyzing landscape preferences. PLOS ONE. 2022;17: e0273519. Available: https://doi.org/10.1371/journal.pone.0273519
