# Supplementary material for: Assessing landscape aesthetic values: Do clouds in photographs influence people’s preferences?
Source: PLoS One. 2023 Jul 28;18(7):e0288424. doi: 10.1371/journal.pone.0288424 (PMC10381034; doi:10.1371/journal.pone.0288424)
Supplement: S7 Table — Significant differences are formatted in bold (sample size: 124 participants). (DOCX) [file pone.0288424.s013.docx]

Table S7: Differences in perceptions for 29 photo pairs with clouds (A) and without clouds (B). Significant differences are formatted in bold (sample size: 124 participants).

| **Paired samples test** | | | | | | | | | | |
| --- | --- | --- | --- | --- | --- | --- | --- | --- | --- | --- |
| Pair | Picture without (B) – with clouds (A) | Paired differences | | | | | T | df | Significance | |
|  |  | Mean | Std. dev. | Std. error | 95% Confidence interval of the difference | |  |  | One-sided p | Two-sided p |
|  |  |  |  |  | Lower value | Upper value |  |  |  |  |
| 1 | 01B - 01A | .146 | 1.150 | .104 | -.059 | .352 | 1.411 | 122 | .080 | .161 |
| 2 | 02B - 02A | .179 | 1.102 | .099 | -.018 | .376 | 1.801 | 122 | **.037** | .074 |
| 3 | 03B - 03A | -.244 | .862 | .078 | -.398 | -.090 | -3.138 | 122 | **.001** | **.002** |
| 4 | 04B - 04A | -.024 | .804 | .073 | -.168 | .119 | -.336 | 122 | .369 | .737 |
| 5 | 05B - 05A | -.260 | 1.253 | .113 | -.484 | -.036 | -2.302 | 122 | **.012** | **.023** |
| 6 | 06B - 06A | -.187 | .978 | .088 | -.362 | -.012 | -2.120 | 122 | **.018** | **.036** |
| 7 | 07B - 07A | .179 | 1.235 | .111 | -.042 | .399 | 1.606 | 122 | .055 | .111 |
| 8 | 08B - 08A | .154 | 1.432 | .129 | -.101 | .410 | 1.197 | 122 | .117 | .234 |
| 9 | 09B - 09A | -.382 | 1.457 | .131 | -.642 | -.122 | -2.908 | 122 | **.002** | **.004** |
| 10 | 10B - 10A | .008 | 1.479 | .133 | -.256 | .272 | .061 | 122 | .476 | .951 |
| 11 | 11B - 11A | -.301 | 1.361 | .123 | -.544 | -.058 | -2.452 | 122 | **.008** | **.016** |
| 12 | 12B - 12A | -.073 | 1.195 | .108 | -.287 | .140 | -.679 | 122 | .249 | .499 |
| 13 | 13B - 13A | -.171 | 1.136 | .102 | -.373 | .032 | -1.667 | 122 | **.049** | .098 |
| 14 | 14B - 14A | -.179 | .869 | .078 | -.334 | -.024 | -2.284 | 122 | **.012** | **.024** |
| 15 | 15B - 15A | -.106 | .885 | .080 | -.264 | .052 | -1.324 | 122 | .094 | .188 |
| 16 | 16B - 16A | -.089 | 1.187 | .107 | -.301 | .123 | -.835 | 122 | .203 | .405 |
| 17 | 17B - 17A | -.057 | 1.089 | .098 | -.251 | .137 | -.580 | 122 | .282 | .563 |
| 18 | 18B - 18A | .122 | 1.053 | .095 | -.066 | .310 | 1.285 | 122 | .101 | .201 |
| 19 | 19B - 19A | .089 | 1.552 | .140 | -.188 | .367 | .639 | 122 | .262 | .524 |
| 20 | 20B - 20A | .154 | .941 | .085 | -.014 | .322 | 1.820 | 122 | **.036** | .071 |
| 21 | 21B - 21A | -.089 | 1.255 | .113 | -.313 | .134 | -.791 | 122 | .215 | .431 |
| 22 | 22B - 22A | -.593 | 1.698 | .153 | -.897 | -.290 | -3.877 | 122 | **<.001** | **<.001** |
| 23 | 23B - 23A | .098 | 1.224 | .110 | -.121 | .316 | .884 | 122 | .189 | .379 |
| 24 | 24B - 24A | .236 | 1.117 | .101 | .036 | .435 | 2.341 | 122 | **.010** | **.021** |
| 25 | 25B - 25A | -.146 | 1.582 | .143 | -.429 | .136 | -1.026 | 122 | .153 | .307 |
| 26 | 26B - 26A | .252 | 1.219 | .110 | .035 | .470 | 2.294 | 122 | **.012** | **.024** |
| 27 | 27B - 27A | .106 | 1.062 | .096 | -.084 | .295 | 1.104 | 122 | .136 | .272 |
| 28 | 28B - 28A | .366 | 1.140 | .103 | .162 | .569 | 3.559 | 122 | **<.001** | **<.001** |
| 29 | 29B - 29A | -.358 | 1.362 | .123 | -.601 | -.115 | -2.913 | 122 | **.002** | **.004** |
