# Supplementary material for: Assessing landscape aesthetic values: Do clouds in photographs influence people’s preferences?
Source: PLoS One. 2023 Jul 28;18(7):e0288424. doi: 10.1371/journal.pone.0288424 (PMC10381034; doi:10.1371/journal.pone.0288424)
Supplement: S8 Table — A: Original picture with clouds, B: manipulated picture without clouds. (DOCX) [file pone.0288424.s014.docx]

Table S8: Mean values and standard deviation (SD) of the variables sky and clouds across picture pairs with decreased (A>B), not changed (A=B), and increased (A<B) preference score. A: Original picture with clouds, B: manipulated picture without clouds.

| **Preference score** | **Sky (%)** | | **Clouds (%)** | |
| --- | --- | --- | --- | --- |
|  | **Mean** | **SD** | **Mean** | **SD** |
| A > B (n = 9) | 33,89 | 10,833 | 17,89 | 17,033 |
| A=B (n =15) | 21,93 | 8,648 | 39,33 | 24,118 |
| A < B (n = 5) | 15,60 | 14,707 | 44,00 | 18,166 |
| Total (n = 29) | 24,55 | 12,158 | 33,48 | 23,176 |
