# Supplementary material for: Assessing landscape aesthetic values: Do clouds in photographs influence people’s preferences?
Source: PLoS One. 2023 Jul 28;18(7):e0288424. doi: 10.1371/journal.pone.0288424 (PMC10381034; doi:10.1371/journal.pone.0288424)
Supplement: S9 Table — Only pictures with significant differences between preference scores of the original and the manipulated picture are included. The spatial shift was determined based on a grid with 9 cells that was overlaid over the pictures (see S6 Fig). (DOCX) [file pone.0288424.s015.docx]

Table S9: Spatial shift hotspots due to cloud removal. Only pictures with significant differences between preference scores of the original and the manipulated picture are included. The spatial shift was determined based on a grid with 9 cells that was overlaid over the pictures (see Fig. S3).

| **Picture** | **Spatial shift of hotspots** |
| --- | --- |
| 2 | no change |
| 3 | from B2 to C1 |
| 5 | from B to A and C |
| 6 | from B2 to A1 |
| 9 | from B2 to A3 |
| 11 | from B to C |
| 13 | no change |
| 14 | from B2 to A1 |
| 20 | from A and C to B |
| 22 | from B2 to A1 |
| 24 | no change |
| 26 | from B2 to C1 |
| 28 | from B2 to C1 |
| 29 | from B to A and C |
