# Supplementary material for: Assessing landscape aesthetic values: Do clouds in photographs influence people’s preferences?
Source: PLoS One. 2023 Jul 28;18(7):e0288424. doi: 10.1371/journal.pone.0288424 (PMC10381034; doi:10.1371/journal.pone.0288424)
Supplement: S10 Table — (DOCX) [file pone.0288424.s016.docx]

Table S10: Main cloud types in the original pictures.

| **Picture** | **Cloud type** |
| --- | --- |
| 1 | cumulus, nimbostratus & cirrostratus |
| 2 | cumulus, cirrus & cirrostratus |
| 3 | cumulus, cirrus & cirrostratus |
| 4 | cumulus |
| 5 | cumulus |
| 6 | cirrocumulus & cumulus |
| 7 | cumulus |
| 8 | cumulus & cirrocumulus |
| 9 | cumulus, cirrus & cirrostratus |
| 10 | cumulus |
| 11 | cumulus & cirrostratus |
| 12 | cumulus, cirrus & cirrostratus |
| 13 | cumulus & cirrostratus |
| 14 | cirrostratus, cirrus & cirrocumulus |
| 15 | cumulus, nimbostratus, cirrocumulus & cirrostratus |
| 16 | cumulus, nimbostratus, cirrocumulus & cirrostratus |
| 17 | cumulus, nimbostratus, cirrocumulus & cirrostratus |
| 18 | cumulus, cirrus & cirrostratus |
| 19 | cumulus |
| 20 | cumulus, nimbostratus, cirrocumulus & cirrostratus |
| 21 | cumulus, nimbostratus & stratocumulus |
| 22 | cumulus & cirrus |
| 23 | cumulus & cirrostratus |
| 24 | cumulus & stratocumulus |
| 25 | cumulus & cirrocumulus |
| 26 | cumulus, cirrocumulus & cirrostratus |
| 27 | cirrostratus, cumulus & cirrocumulus |
| 28 | cumulus & cirrocumulus |
| 29 | cumulus & cirrocumulus |
