# Supplementary material for: Assessing landscape aesthetic values: Do clouds in photographs influence people’s preferences?
Source: PLoS One. 2023 Jul 28;18(7):e0288424. doi: 10.1371/journal.pone.0288424 (PMC10381034; doi:10.1371/journal.pone.0288424)
Supplement: Attachment — Submitted filename: Peer reviewer feedback - PONE-D-23-07973.docx [file pone.0288424.s018.docx]

**Title**

Do clouds in landscape photographs influence people’s preferences?

**Summary**

The authors present a study examining how clouds, and a host of other independent variables, might affect preference ratings for landscape views. Results indicate differences between images with and without clouds, and according to the proportion of sky depicted. The paper attempts to relate these findings to a cultural ecosystem services framework.

**Overall comments**

Whilst I endorse the authors’ focus on this overlooked component of landscape evaluation, in its current form the paper does not provide adequate information about the study design or its participants, and reporting of results requires substantial additional information.

The introduction, and materials and methods, should be improved to help the reader understand the visual stimuli being tested and the demographic characteristics of participants. The limited information presented here renders the study impossible to reproduce.

The results are difficult to follow and interpret, and seem to combine empirical findings with visually inferred observations. This section needs careful rewriting to present the results in a way that readers can both understand and suitably interrogate.

The authors must also consider publishing their image sets, analysis scripts, and raw data on the Open Science Framework (or similar platform) such that their findings can be reproduced.

**Specific comments**

#1 The intentions of the study could be clearer in the abstract. For example at line #13 “*Usually, pictures with similar weather and light conditions (i.e., blue sky) are used to reduce the potential influence on landscape preferences*” should be rephrased to explain what potential confounders ‘blue sky’ conditions are mitigating.

#2 The introduction would benefit from further information on the different forms clouds can take. The example photographs included in the paper depict various types of cumulus clouds, were these the only cloud formations included across the images used? For example, participant responses to cirrus stratus or altocumulus may have varied substantially compared to cumulus clouds. Moreover, why did the authors choose the cloudscapes included in this study over other formations?

#3 An overview of the methods typically used to assess landscape preferences in photographs would help the reader understand how aesthetic value is usually measured, and therefore how this study's methods might be applicable to other work in this field.

#4 The discussion on study design should include an explanation of how the authors arrived at a suitable sample size. Was a power calculation performed before recruitment? What sample sizes are common in other studies that assess landscape aesthetics?

#5 What were the characteristics of the participants? The authors note that the survey was distributed to undergraduates and their family members, but no information is provided on who actually took part and therefore who their findings relate to. Please include a breakdown of participant demographics, with n’s for each grouping specified.

#6 The authors note that the survey took 5-10 minutes to complete (line #102). This description would benefit from further details. Where did participants complete the study, at home or in a controlled laboratory setting? On what kind of device? How were the images displayed and for how long? Was completion time monitored, and any quality control in place to prevent people from ‘speeding’ through without paying attention? Were participants primed as to the purposes of the study? Virtual eye tracking was used but few details are provided on how and why this method is appropriate, how does it compare to actual eye tracking? On this point, the authors simply refer to “*Schirpke et al.*” but I suggest that the reader should be presented with these points without having to refer to another study – were the elements of that study followed exactly? How do the methods here deviate?

#7 At line #108 the authors refer to “*two former surveys*” which the reader assumes are those referenced previously. If this is the case, it should be clarified which studies these are with appropriate referencing and details where necessary. For example, why is it appropriate to compare preference scores from this study to those mentioned?

#8 At line 152 the authors note that “*areas hidden by vegetation, buildings, or mountains, were excluded from further analysis.*” The reader is curious why it is appropriate for these potential confounding elements to be omitted from the analysis. The authors should provide justification for this approach in the manuscript.

#9 Why it is acceptable to replace missing values with mean values (line #167)? Did this method apply to all IVs? Could the authors explain this in a way such that a reader who is unfamiliar with statistical methods can understand the steps taken.

#10 At line #176 the authors mention tests for normality but do not provide results from these tests until line #206. I suggest clarifying these outcomes here or at least acknowledging that tests were passed and reported later.

#11 To aid interpretation of the results, Figure 2 would be clearer if both A and B images were shown for each example. At present the comparisons being assessed are not clear.

#12 Tabular data underpinning Figure 3 should be displayed. Importantly, what manipulation to the data has been applied to create this figure? Are these mean preference scores across groupings? Are these differences across groups statistically significant? If so, what test has been applied to determine this relationship?

#13 Furthermore, it is not clear how the percentages reported in line #196 have been deduced. Further clarification is required to help these results be interpreted and reproduced.

#14 Table 2 is difficult to interpret in its current form since IVs have been described by their coded descriptors (which the reader can only interpret by cross referencing with Table S1 in supplementary materials). I suggest these are replaced with more easily interpretable labels, for example, “*I_HP_1..n_ (%)*” could read “*Sky within hotspots (%)*”. Furthermore, significant coefficients in table 2 might be highlighted in bold to improve readability.

#15 Figure 4 shows some intriguing patterns, but I found it very difficult to follow the descriptions provided in lines #212 to #230. Are the authors referring to results from table 2? If so, it would be useful to report coefficients and p-values in these descriptions so the reader can understand the relationships being described. More importantly, these descriptions appear to refer to images grouped according to whether they do or do not contain clouds. Were two regression models run based on these categories? The IV in the table simply refers to % cloud cover, and clouds Yes/No as a binary variable is not included nor as an interaction term. Greater clarification is required on how the authors can qualify statements such as “*Pictures with clouds that had higher preference scores comprised on average more hotspots*” and “*preferences for landscapes without clouds were often related to the presence of a large top hotspot*”. Have these been empirically tested or visually inferred from the data?

#16 At line #220 the authors suggest that “*a high proportion of background (EZ4_P1..n) also reduced the negative effects of anthropogenic structures in the foreground (Eart_P1..n)*”. How was this dependency tested? No interaction effects between IVs are reported.

#17 Likewise, at line #299 the authors report that “*the hotspots in the more attractive image pair are located more towards the sides than in the center.*” Was this empirically tested or is this an observation based on one or more photos?

#18 I am unclear on how the 40% threshold for cloud cover noted in line #246 has been derived. The discussion section should not be the first place results are reported.

#19 More importantly, and in relation to comment #2, nimbostratus clouds are mentioned in line #247, and single cumulus clouds mentioned in line #249. This is the first time cloud type has been introduced and suggests substantial heterogeneity in the clouds depicted. More information is required on the composition of these cloud forms in the introduction and materials and methods, how has this been accounted for as a potential confounding factor?

#20 The authors present a quantitative analysis of how clouds might impact preferences for landscape scenes, and attempt to relate this to cultural ecosystem services. However, as hinted at by the phrase ‘cultural’, these values can be highly subjective. It would be valuable to the reader to understand the limitations of this approach, particularly with respect to the fertile literature on subjective vs objective aesthetic assessments.
