# Supplementary material for: Assessing landscape aesthetic values: Do clouds in photographs influence people’s preferences?
Source: PLoS One. 2023 Jul 28;18(7):e0288424. doi: 10.1371/journal.pone.0288424 (PMC10381034; doi:10.1371/journal.pone.0288424)
Supplement: Attachment — Submitted filename: Eyetracking-sky_responses2.docx [file pone.0288424.s020.docx]

**Reviewer #1:**

This paper has been improved with revision. I have only a few remaining comments to further strengthen or clarify the work.
Response: Thank you for your time and effort in re-reviewing our manuscript and your positive feedback. We have also now addressed all additional comments.

139 I acknowledge that there is no set scale for assessing landscape preferences, and this may be an issue of translation, but I am finding least preferred and most preferred somewhat nonsensical for photos explored individually. Least and most are comparative terms. Could the authors check the best translation, if formal equivalence exists, as I’m sure this must have made more sense in German.

Response: Thank you for pointing this out. Indeed, there was no comparison among the pictures, but we asked the people to indicate how much they liked the pictures on a scale from 1 = ‘I don’t like it at all’ to 10 = ‘I like it very much’. We now corrected the description in the manuscript.

I’m finding the ‘intensity of visual elements’ variable a bit tricky to conceptualize, and indeed quite a few of the eye-tracking variables are a bit hard to conceptualize. Is it density or concentration or diversity? The Table 1 isn’t always helpful, given that the units are mostly described as percentages but the descriptions include things like areas and contributions to probability.

Response: 3M-VAS describes “intensity” with “luminance contrast, brightness, black/white contrast”. We added this explanation to the description in Table 1. The variables are expressed in %, as it indicates the overall contribution to the probability of looking at a hotspot. Accordingly, we refined the description of these variables in Table 1.

235-238 is not easy to understand and I think these steps should be understandable without looking at the Schirpke reference cited.

Response: We revised the description of the methodological steps by adding all details. We also added an additional figure to the supplementary material (new Fig. S5), which illustrates the estimation of the contribution of the visual elements.

Some of the results could be clearer if some additional terms could be added, for instance in line 311 add “with clouds” after “lower”, or in line 323, add “in the” before “pair” (if I’m interpreting correctly). Additionally 325 add “with cloud removal after “photos” at the end of that line.
At the end of 345 you indicate a high intensity of visual elements was associated with pictures without clouds. I can’t seem to find the supplemental data reporting this. I see the difference score regression. Maybe include the relevant stats in the body and clearly note where reported where those models can be found.

Response: We added the suggested terms or rephrased the sentences to improve clarity. The sentence in line 345 still referred to Table 2, where the results of the regression analysis are reported. We now added the cross-reference to this table to clearly indicate the underlying data. Moreover, we added another explanation in the table caption to clearly indicate the meaning of the coefficients; i.e., a negative regression coefficient B indicates that this variable has a positive effect on the preference score of photos with clouds.

Discussion starts with reporting on a hypothesis that I don’t remember being declared earlier.
Response: We rephrased the sentence to match our hypothesis presented at the end of the introduction.

Small things but still worth mentioning.
80-81 This sentence seems tautological, maybe drop the first part

Response: We removed the first part of the sentence and slightly rephrased the second part.

89-93 Given the paragraph that comes before it seems unclear, but somewhat likely that clouds will alter preferences. This paragraph doesn’t seem to acknowledge the one before. It could also be done by noting that it is not just “attracting attention” that affects preferences but the emotional impact and mood that clouds can bring to an image.
Response: Thank you for this suggestion. We now also indicate that preferences are influence by emotions and moods.

143 use “assessed” rather than obtained
Response: Done.

156 Is the estimated time to complete the researchers’ estimation or based on actual completion?
Response: We could not record the time of the participants to complete the questionnaire. However, we measured how long it took us to fill it out. We rephrased the sentence to clarify that we estimated the time.

402 using “were” reads as if you found it rather than Svobodova. Maybe “have been”?
Response: Done.

433 and elsewhere Using “percentage sky” reads overly technical for discussion. Percentage “of” sky

Response: We changed to “percentage of sky” throughout the manuscript.

**Reviewer #2:**

I appreciate the effort you have put in revising the manuscript answering all the comments from authors.
I only have one small doubt to clarify,

Response: Thank you for your time and effort in re-reviewing our manuscript and your positive feedback. We have also now addressed all additional comments.

Starting from line 138 - You have mentioned that ....... to decide whether their preference is positive or negative. But you have included the likert scale as 1 - least preferred (which is the lowest level of preference and obviously not a negative response). Please explain this incase I misunderstood!

Response: Indeed, the description of the rating scale was wrong, as we asked the people to indicate how much they liked the pictures on a scale from 1 = ‘I don’t like it at all’ to 10 = ‘I like it very much’. In this case, values >= 5 can be interpreted as positive and below 5 as negative. We now corrected the description of the rating scale in the manuscript.

Line 209 - 3M-WAS

Response: Corrected.

Line 213 - ...... their eyes on in the initial..... ???

Response: We revised the sentence to clarify the meaning.

Other than that all the comments are addressed. Great work.

Response: Thank you.

**Reviewer #3:**

I commend the authors on their revised manuscript. They have addressed all reviewer comments and the resulting paper is much improved and offers a valuable contribution to the field.
I have just one comment I think it would be worth addressing before recommending publication:
Response: Thank you for your time and effort in re-reviewing our manuscript and your positive feedback. We have also now addressed all additional comments.

The manipulated images are, in some cases, very obviously edited. The blue sky has little texture, and the lighting of the foreground landscape does not match that provided by a typical blue sky. It would be valuable to mention this limitation, and its possible effects on ratings; could participant ratings have reflected (at least partly) negative reactions to Photoshopped scenes?

Response: We agree that this could have been partly influenced the preferences scores. We now added this limitation to the discussion section.
